# Homologous Recombination Defective *Arabidopsis* Mutants Exhibit Enhanced Sensitivity to Abscisic Acid

**DOI:** 10.1371/journal.pone.0169294

**Published:** 2017-01-03

**Authors:** Sujit Roy, Kali Pada Das

**Affiliations:** Protein Chemistry laboratory, Department of Chemistry, Bose Institute, Kolkata, India; Tulane University Health Sciences Center, UNITED STATES

## Abstract

Abscisic acid (ABA) acts as an important plant hormone in regulating various aspects of plant growth and developmental processes particularly under abiotic stress conditions. An increased ABA level in plant cells inhibits DNA replication and cell division, causing plant growth retardation. In this study, we have investigated the effects of ABA on the growth responses of some major loss-of-function mutants of DNA double-stand break (DSB) repair genes in *Arabidopsis* during seed germination and early stages of seedling growth for understanding the role of ABA in the induction of genome instability in plants. A comparative analysis of ABA sensitivity of wild-type *Arabidopsis* and the knockout mutant lines related to DSB sensors, including *atatm*, *atatr*, the non-homologous end joining (NHEJ) pathway genes, and mutants related to homologous recombination (HR) pathway genes showed relatively enhanced sensitivity of *atatr* and HR-related mutants to ABA treatment. The expression levels of HR-related genes were increased in wild-type *Arabidopsis* (Col-0) during seed germination and early stages of seedling growth. Immunoblotting experiments detected phosphorylation of histone H2AX in wild-type (Col-0) and DSB repair gene mutants after ABA treatment, indicating the activation of DNA damage response due to ABA treatment. Analyses of DSB repair kinetics using comet assay under neutral condition have revealed comparatively slower DSB repair activity in HR mutants. Overall, our results have provided comprehensive information on the possible effect of ABA on DNA repair machinery in plants and also indicated potential functional involvement of HR pathway in repairing ABA induced DNA damage in *Arabidopsis*.

## Introduction

Plants are frequently exposed to various environmental stress conditions and therefore have evolved with highly complex and elaborated mechanisms for integrating different stress signals and respond to them in a coordinated and comprehensive manner to adjust and survive under such stress conditions. Plants respond to abiotic stresses predominantly via the rapid changes in the expression levels of the stress responsive genes at the transcriptional level and by means of accumulation of metabolic compounds, which help in stress adaptation. Abiotic stresses, such as UV-radiation, high soil salinity, water deficit, low temperature, nutrient deficiencies, herbicide treatment and heavy metal toxicity frequently induce the accumulation of the phytohormone abscisic acid (ABA) in plants [1,2,3]. ABA plays key roles in regulating various fundamental aspects of plant growth and development throughout the life span [4]. Multiple evidences from previous molecular genetic studies using ABA deficient *Arabidopsis* mutants have demonstrated role of ABA in controlling the normal growth pattern in plants. In contrast, exogenous application of ABA inhibits cell division and causes plant growth retardation [5,6,7]. Genetic and molecular studies have helped in the identification and characterization of several genes involved in ABA responses in plants [8,9].

The generation of reactive oxygen species (ROS) by the activity of membrane bound NADPH oxidases [10], as a consequence of exposure to abiotic stresses, has been considered as one of the major reasons of ABA mediated plant growth inhibition. High intracellular level of ROS induces oxidative stress via damage to most fundamental macromolecules, including lipids, proteins, and nucleic acids, resulting in cell disruption and inhibition normal pattern of plant growth and development [11,5,7]. ROS induces various forms of damages in the double helical structure of DNA. However, 7, 8-dihydro-8-oxoguanine (8-oxo-G) has been considered as one of the predominant mutagenic lesions in DNA, produced via oxidative damage [12]. During DNA replication 8-oxo-G mispairs with adenine, generates GC/TA transversions, thus inducing mutagenesis. Other forms of oxidative DNA damage, such as 1,2-dihydro-2-oxoadenine (2-OH-A) inhibits DNA replication and therefore activates the cell cycle checkpoint functions to arrest cell cycle progression via the activity of ATR (Ataxia telangiectasia and Rad3 related) kinase and SOG1 (Suppressor of gamma response 1), a plant specific transcription factor. The oxidative damages in the DNA are mainly repaired via the base excision repair (BER) pathway during S phase of the cell cycle to eliminate the mutagenic effects. However, prolong replication stress due to error-prone or inefficient BER activity generates collapsed replication forks and frequently encourages the formation of other serious forms of DNA damages, including the single stand breaks (SSBs) and double-strand breaks (DSBs) [13], respectively. Lack of efficient repair of SSBs and particularly DSBs may induce structural abnormalities in chromosomes, which in turn significantly affect plant growth and development during early germination stages and seedling growth due to inhibition of DNA replication and transcriptional processes.

Double-strand breaks (DSBs) in DNA double helix is considered as one of the major forms of DNA damage [14]. DSBs are frequently induced by genotoxic stresses. However, error-prone DNA replication and trans-lesion synthesis (TLS) of collapsed replication forks also induce the generation DSBs [15]. Unrepaired DSBs in the genome cause deletion of chromosome fragments and thus genome instability and even cell death in extreme cases, indicating the importance of rapid detection and efficient repair of DSBs for maintaining genome stability [16]. Two fundamental mechanisms, namely the homologous recombination (HR) and the non-homologous-end joining (NHEJ) pathways are involved in repair of DSBs [17,18]. The HR pathway is based on the activity of RAD52 epistasis groups of proteins, such as RAD51, RAD52, RAD54, RAD55, RAD57 and the MRN complex, comprising of MRE11, RAD50 and NBS1. HR pathway operates mainly during the S and G2 phases of cell cycle and utilizes the intact copy of the homologous DNA as template for repairing the damaged strand to restore the original sequence with high fidelity [19,20]. HR acts as the predominant form of DSB repair pathway in bacterial and yeast cells, however, it plays key role in recombination repair in meiotic cells in plants. In somatic plant cells, DSBs are mainly repaired via the NHEJ pathway during G1 to early S-phase of the cell cycle [21,22]. However, unlike the high fidelity HR mediated repair, NHEJ is known as an illegitimate pathway as the broken DNA ends are joined directly via this pathway, without considering the sequence homology [21,17]. NHEJ repair pathway recruits the activity KU70/80 heterodimer for the detection of DSBs, followed by the gap filling DNA synthesis and rejoining of DNA ends by the activity of DNA polymerase λ and DNA ligase4-XRCC4 (X-RAY CROSS COMPLEMENTATION PROTEIN4) complex, respectively [22,23].

Much of the earlier studies have been focused on ABA-mediated rapid physiological responses in the context of short term stress resistance in plants. However, information is still inadequate about whether ABA or ABA signaling affects genome stability in plants. Understanding this mechanism might have relevance for the environmental stress tolerance in plants. In addition, although previous studies have investigated the effects of ABA on DNA replication machinery and progression of cell cycle [4,24], influence of this stress hormone on the components of plant DNA damage repair system has not been studied in detail. To address this issue, in this study, we have utilized knockout mutant lines of some major genes involved in DSB sensing and repair in *Arabidopsis* via the HR and NHEJ pathway and along with wild-type *Arabidopsis* (Col-0), systemically investigated the growth responses of such mutant lines under ABA stress during seed germination and early stages of seedling growth to understand the effect of ABA in the induction of genome instability and the possible mechanism of repair of DNA strand breaks induced by ABA. Our results on growth response studies on DSB-related mutants, the steady-state levels of the major DSB-related genes in *Arabidopsis* under ABA stress and the DSB repair kinetics have indicated importance of HR pathway in repairing ABA-induced DNA damage in plant genome.

## Materials and Methods

### Plant materials and growth conditions

The *Arabidopsis thaliana atku80* (Salk_016627C), *atku70* (salk_123114C), *atlig4* (Salk_044027C), *atxrcc4* (Salk_052736C), *atpol*λ*-1* (Salk_075391C), *atmre11-3* (Salk_054418), *atnbs1* (Salk_057446C), *atrad50* (Salk_084967), *atrad51* (CS303003), *atrad52* (Salk_089362), *atrad54* (Salk_038057C), *atatr* (Salk_032841), *atbrca1* (Salk_014731), respectively are in the Columbia accession. The *atku80*, *atku70*, *atlig4*, *atxrcc4*, *atpol*λ*-1*, *atmre11-3*, *atnbs1*, *atrad50*, *atrad51*, and *atatr* T-DNA knockout lines were received from the Arabidopsis Biological Resource Centre, Ohio [25]. The *atm-2* homozygous T-DNA-insertion mutant line (in Wassilevskija background) was obtained from Prof. A. B. Britt, University of California Davis, USA. The *atbrca1* homozygous T-DNA-insertion mutant line (Salk_014731) was obtained from Prof. Holger Puchta at the Botanisches Institut II, Universita¨t Karlsruhe, Karlsruhe, Germany. *Arabidopsis thaliana* seeds (Col-0, Ws and other T-DNA insertion mutant lines) were surface sterilized and grown on Murashige and Skoog medium (MS) containing 0.8% Bactoagar and 1% sucrose, respectively. Seeds were germinated and seedlings were grown by essentially following the conditions described earlier [23].

### Characterization of T-DNA insertion mutant lines

The homozygous T-DNA insertion mutant lines *atpol*λ*-1* (Salk_075391C), *atm-2* (Salk_006953) and *atbrca1* (Salk_014731) were described previously [23,26,27]. For identification of the homozygous mutant lines of other damage repair pathway genes, the T-DNA insertion mutant lines, either homozygous or heterozygous for the respective alleles were subjected to PCR genotyping analyses. Individual plants were then checked by PCR using the left border specific primer LBb1.3 (5’ATTTTGCCGATTTCGGAAC3’) and the gene specific primers LP and RP for *AtKU80*, *AtKU70*, *AtLig4*, *AtXRCC4*, *AtPol*λ, *AtATR*, *AtMRE11 AtNBS1*, *AtRAD50*, and *AtRAD51*, respectively (S1 Table).

### ABA treatment, germination assay and quantitative plant growth measurement

Surface sterilized seeds (about 75 seeds) were grown on MS medium containing different concentrations of ABA. The germination rates and post-germination growth of the seedlings were calculated. Seed germination rate was analysed three days after placing the plates on culture rack. The experiment was repeated at least three times with three plates in each case. For post-germination growth analysis, 7-days-old pre-germinated seedlings of different genotypes were transferred onto MS-agar plates supplemented with increasing concentrations of ABA. Plants were maintained under 16 h light/8 h dark photoperiodic cycles. After 7 days, relative ABA sensitivity of wild-type (Col-0, Ws) and mutant lines were compared by measuring the hypocotyl lengths, primary root growth, number of lateral root formation and relative fresh weights of the seedling, respectively. The experiment was repeated at least three times, using 75 seedlings in three replicates in each time. For *atm-2* homozygous mutant line, growth responses and other parameters were compared with Wassilevskija (Ws) background. However, except for flowering time, comparable results were obtained for the two wild-type accessions, Col-0 and Ws and thus data from Ws background has not been included in the results. Initial standardization of ABA concentrations for treatment during seed germination and early stages of seedling growth was performed by examining the phenotypic responses of wild-type (Col-0) *Arabidopsis* and other selected mutants in presence of different concentrations of ABA. For wild-type (Col-0) *Arabidopsis*, although germination was compromised, seeds germinated even in presence of up to 5 μM ABA, while DSB related gene mutants showed decreased germination response in presence of 1 μM ABA and beyond. On the other hand, for seedling treatment, whereas wild-type (Col-0) *Arabidopsis* seedlings were found to tolerate relatively higher concentrations of ABA (120–150 μM), consistent with the previous studies [5], DSB repair gene mutants displayed severe growth retardation beyond 60 μM ABA when treatment was given to 7-days-old pre-germinated seedlings. Based on this, germination performance assay was carried out in presence of less than 1 μM concentration of ABA (0–0.5 μM). For post-germination growth response studies, up to 50 μM ABA concentration was used since up to this concentration comparative growth response study was feasible.

### Analysis of transcript profile by quantitative real-time PCR

Transcript profiles of DSB-related genes were analysed by quantitative real-time PCR by following the method described previously [28] with minor modifications. For ABA-mediated gene expression analysis during seed germination, surface sterilized wild-type (Col-0) *Arabidopsis* seeds were germinated in MS-agar medium in the absence or presence of 0.5 and 5 μM ABA, respectively. Germinated seedlings were maintained in a growth chamber with 16-h-light/8-h-dark photocycles. 5-days-old seedlings were harvested for RNA isolation and subsequent real-time gene expression analysis. To examine the ABA mediated expression pattern of DSB related genes during early stages of seedling growth, surface-sterilized wild-type (Col-0) *Arabidopsis* seeds were grown on MS medium (containing 0.8% Bactoagar and 1% Sucrose), transferred to a growth chamber with 16-h-light/8-h-dark photocycles, and then 7-days-old pre-germinated seedlings were transferred to one-half-strength liquid MS medium containing 30 μM ABA. Seedlings were collected after incubation for various time points for RNA isolation and transcript profile analysis. Total RNA was isolated from approximately 100 mg of seedling tissue by using Trizol reagent (Invitrogen) following manufacturer’s instructions. The RNA samples were then treated with DNase I (Ambion) following manufacturer’s protocol. Approximately 0.75 μg of total RNA was used in a 10 μL reaction for reverse transcription with oligo (dT_20_) primers and SuperScript III reverse transcriptase (Invitrogen) following manufacturer’s instructions. RT reactions were 5-fold diluted and 3 μL from the dilutions were used as template for PCR. The real-time PCR amplification was carried out using iQ SYBR Green Supermix (Bio-Rad) in 15 μL reactions with three technical repeats. The PCR cycle conditions included: 94°C for 2 min, followed by 40 cycles of 94°C for 15 s, 30 s at 60 to 65°C s for 30 s and 60°C for 30 s, respectively. Three biological replications were performed for each experiment. The values indicate averages of triplicate assays for each reverse transcribed sample. The primers and detail annealing temperatures used for real-time PCR have been summarized in S2 Table.

### Analysis of induction and repair of DSBs by neutral comet assay

The extent of ABA-induced nuclear DNA damage in wild-type and DSB repair related gene mutant lines were detected by analyzing the accumulation of double-strand breaks using neutral comet assay [29,30]. The level of DNA damage was measured and represented as the percentage of DNA detected in comet tail in comparison with the total amount of DNA present in the head and tail of the comet, respectively. Data were collected from at least three independent experiments and comet evaluation, statistical analysis and calculation of remaining percentage of DNA were carried out following the method described earlier [29,30].

### Extraction of histone protein

Histone protein was extracted from *Arabidopsis* seedlings by following the procedures described previously [31,32] with some modifications. Protein extraction was carried out in presence of sodium fluoride (Sigma) and sodium ortho-vanadate (Sigma) as protein phosphatase inhibitor at the final concentrations of 30 and 100 mM, respectively. Protein concentrations in the samples were determined following Bradford assay with bovine serum albumin (BSA) as standard (Fraction V, Sigma).

### Immunoblotting

Approximately 60 μg of protein samples were separated on a 15% sodium dodecyl sulphate-polyacrylamide gel and electro-transferred onto a polyvinylidene fluoride (PVDF) membrane (Bio-Rad). Phosphorylation of histone H2AX (γ-H2AX) in the protein extracts were detected by immunoblotting following by essentially following the method described previously [32]. Blots were incubated in presence of rabbit anti-plant γ-H2AX polyclonal antibody (1:1000 dilutions) in 3% nonfat milk in 1X TBS-T for overnight at 4°C. Alkaline phosphatase conjugated anti-rabbit IgG (Sigma) was used as secondary antibody (1:10000 dilutions) for the detection of innumo-reactive bands on the membrane by following the alkaline phosphatase activity assay using BCIP ⁄NBT system as substrate (Sigma) [33].

### Statistical analysis

The ANOVA software Statstica 6.0 was utilized for statistical analysis of the results for germination performance and other morphological parameters of wild-type and mutant lines. In comet analysis, Student’s t-test was used to measure the extent of accumulation and repair of DNA double-strand breaks generated in *Arabidopsis* seedlings due to ABA treatment. Statistical probability ≤ 0.05 was considered as significant.

## Results

### DSB-related mutants display ABA-sensitive growth response

Evidences from previous research have demonstrated ABA mediated induction of genotoxic stress in plants [34]. Various abiotic stress responses are known to be regulated by ABA. Earlier studies have indicated role of ABA in the induction of DNA double-strand breaks (DSBs) in plant genome and enhancement of rate of homologous recombination in wild-type *Arabidopsis* and an ABA overly sensitive mutant, *abo4-1* [5]. Various environmental stress factors and endogenous processes, which impose genotoxic stress and inhibition of DNA replication, may lead to collapsed replication fork and error-prone DNA repair, resulting in DNA single and double-strand breaks (SSBs and DSBs) and finally genome instability. Therefore, recognition of DNA damage is the key step and includes different mechanisms for initiating the DNA damage response signaling, activation cell-cycle check point functions, transcriptional response and recruitment of specific damage repair pathways.

In plant somatic cells, DSBs are mainly repaired via non-homologous end joining (NHEJ) mechanisms. However, the HR pathway also plays an important role in repairing plant genome in an error-free manner [22,35]. To determine the role of ABA in the induction of DSBs and genome instability in plants and to investigate the possible mechanism of repair, we selected the major marker genes functioning in DSB detection, signaling and repair processes. The complex of KU70 and KU80, the core component of NHEJ pathway, plays key role in DSB detection in plants [21]. DNA ligase 4-XRCCA4 complex and Pol λ are functional in processing broken DNA ends and repair synthesis in NHEJ mediated DSB repair [23,36]. MRE11, RAD50 and NBS1, the three components of the multifunctional MRN complex, are essential components in DSB signaling and detection and also involved in DSB repair via HR pathway [36,37]. RAD51 (RADiation sensitive51), homologue of bacterial RecA recombinase, is one key components of homology dependent DSB repair (HR) pathway [38]. RAD52 (RADiation sensitive52) also plays key role in homology-dependent DSB repair and recombination [39]. RAD52 mediates RAD51 loading onto single-stranded DNA ends, facilitating initiation of homologous recombination and catalyzing DNA annealing [40]. RAD54 is a member of RAD52 epistasis group of proteins and belongs to the SWI2/SNF2 family of DNA-stimulated ATPases. RAD54 plays crucial role in HR mediated DSB repair, both as a chromatin remodelling factor and as the mediator of the RAD51 nucleoprotein filament [41]. BRCA1 (breast cancer susceptibility 1), together with RAD51 and BARD1 (BRCA1-associated RING domain protein 1), plays vital role in HR-mediated DSB repair [27]. The phosphoinositide-3-kinase-related protein kinases (PIKKs), ATM and ATR functions as master regulators of DNA damage response pathway, regulating cell-cycle progression and activation of DNA repair pathways in response to genotoxic stress (ATM kinase) and replication blockage (ATR kinase), respectively [36,42]. The T-DNA insertion mutants (loss-off-function mutant lines) of such selected DSB signaling and repair genes in *Arabidopsis* were isolated and characterized (Supplementary material, S1 and S2 Figs) to confirm the loss-off-function mutant lines of the corresponding DSB sensor/signaling (*atatm-2*, *atatr*, *atmre11*, *atrad50 and atnbs1*), NHEJ (*atku80*, *atku70*, *atpol*λ*-1*, *atlig4* and *atxrcc4*) and HR (*atrad51*, *atrad52*, *atrad54 and atbrca1*) pathway genes in *Arabidopsis*. We next compared the relative ABA-sensitivity of the homozygous mutant lines with wild-type (Col-0) *Arabidopsis*. The phenotypic responses of wild-type and DSB-related mutant lines were analysed in presence of different concentrations of exogenously applied ABA during seed germination and post-germination seedling growth.

**Fig 1:**
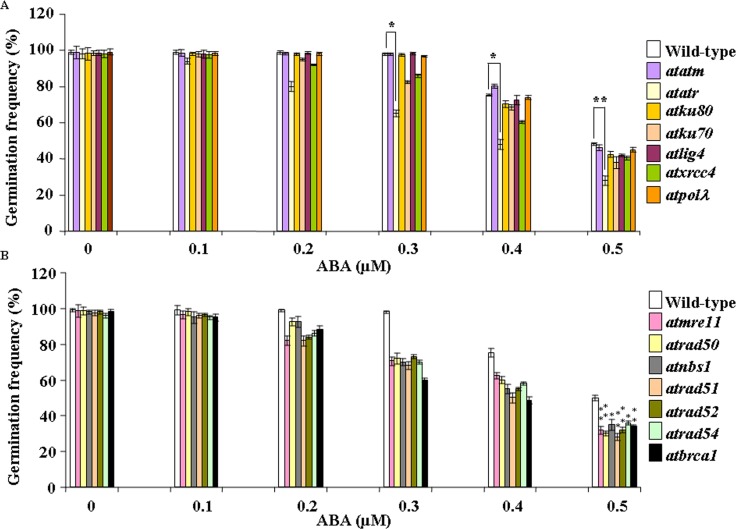
Comparative analysis of ABA sensitivity of wild-type *Arabidopsis* and DNA double-strand break (DSB)-repair related mutants in presence of exogenously applied abscisic acid during seed germination. *Arabidopsis* seeds from the wild type (Wt Col) and DSB-related mutants were germinated on MS-agar medium containing different concentrations of ABA. Plates were kept in the dark at 4°C for 3–5 days and then transferred to light chambers maintained at 22°C with 16-h-light/8-h-dark periods, respectively. One-week-old plants were tested for their sensitivity. (A) Satatistical analyses of germination rates in wild-type and DSB-related mutant plants grown in the absence or presence of increasing concentrations of ABA. (B) Similar experimental conditions were used as in (A) for analyzing the germination rates of wild-type and other DSB-related mutant lines. The bars represent mean values of from three independent observations. *P < 0.05, **P < 0.01 relative to respective controls (n = 3). In each case approximately 75 seeds were counted.

Seed germination rates of wild-type *Arabidopsis* and DSB-related mutant lines were first compared by growing seeds on MS-agar medium containing various concentrations of ABA. Under control condition, any difference in germination rates could be hardly detected in wild-type (Col-0) and other mutants (Fig 1A and 1B). As expected, germination rate was decreased in presence of increasing concentrations of ABA in both wild-type *Arabidopsis* and other DSB-related mutants (Fig 1). Whereas, germination response was prominently affected in presence of 0.4 and 0.5 μM ABA, only marginal response was observed at lower concentrations of ABA for both wild-type *Arabidopsis* and other DSB-related mutants. The relative ABA sensitivity of wild-type and HR-related mutants could be detected even at 0.3 μM ABA (Fig 1A). As compared with control condition (without ABA), in presence of 0.5 μM ABA, germination rates were decreased to approximately 51% in wild-type *Arabidopsis*, about 53.5% in *atatm-2* (~1.0-fold less than wild-type) and 71% in case of *atatr* (~1.7-fold less than wild-type), respectively (Fig 1A) (*P*<0.2–0.01). For the ‘classic’ NHEJ-related mutants, as compared with untreated control (0 μM ABA), germination rates were decreased to approximately 57% in *atku80*, 61.2% in *atku70*, 57.1% in *atlig4*, 59% in *atxrcc4* and 54% in *atpol*λ, respectively (*P*<0.01–0.03) (Fig 1A). This was corresponding to ~1.14, 1.26, 1.14, 1.2 and 1.06-fold reduction in germination rates for the respective mutants than wild-type (Col-0) at 0.5 μM ABA. On the other hand, as compared with untreated control, in the HR-related mutants, seed germination rates were declined to approximately 67.3% in *atmre11*, 69.3% in *atrad50*, 64.2% in *atnbs1*, 71.1% in *atrad51*, 67.3% *atrad52*, 62.5% in *atrad54* and 65.3% in *atbrca1* in presence of 0.5 μM ABA (Fig 1B) (*P*<0.001–0.008), indicating ~1.3–1.6-fold reduction in germination rates in HR mutants than wild-type at this concentration of ABA (*P*<0.01–0.03). Furthermore, as compared with wild-type and NHEJ-related mutant lines, root growth assay also showed relatively higher ABA-sensitivity of HR-related mutants and *attar* (data not shown). Taken together, these results have indicated relatively higher ABA sensitivity of *Arabidopsis* HR pathway mutants than NHEJ mutant lines.

### Post-germination growth responses DSB-related mutants following exogenous ABA treatment

**Fig 2:**
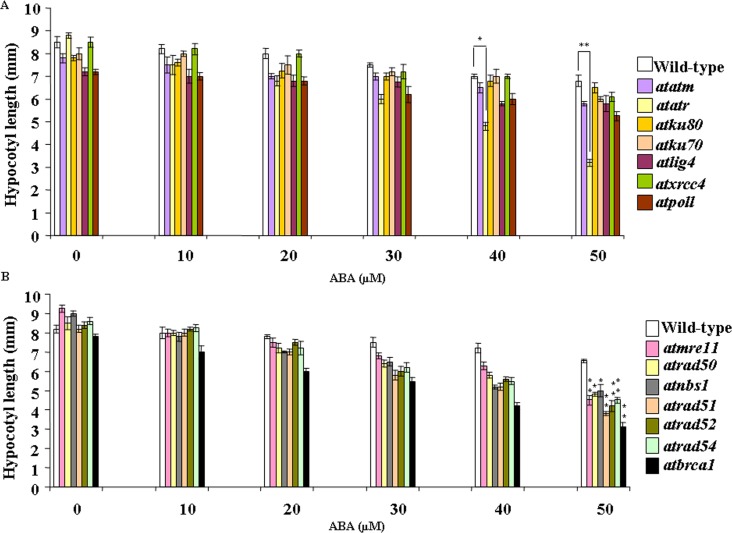
Growth responses of DSB-related mutants in presence of increasing concentrations of ABA during early seedling stage. 7-days-old pre-germinated wild-type (Wt-Col) and DSB-related mutant seedlings were transferred to MS medium containing different concentrations of ABA and maintained under 16 h light/8 h dark photoperiod for another 7-days and then growth responses were analysed. (A) and (B) Satatistical analyses of hypocotyl length of wild-type and mutant seedlings grown in absence or presence of increasing concentrations of ABA. Data shown are means ± SD of three independent replications. At least 75 seedlings were counted in each time. *P < 0.05, **P < 0.01 relative to respective controls (n = 3).

To determine the relative ABA-sensitivity of DSB-related mutants during early stages of seedling growth, 7-days-old wild-type and other DSB-related mutant seedlings, germinated and grown on MS-agar medium, were transferred to MS-medium containing different concentrations of ABA. Seedlings were grown for another 7 days and the relative growth responses of the seedlings were analysed. Seedling growth was affected in both wild-type *Arabidopsis* and DSB-related mutants (Fig 2). The elongation of hypocotyl length was inhibited in a dose dependent manner in wild-type and other mutant lines. As compared with untreated control seedlings, inhibition of hypocotyl length was approximately 1.2-fold in wild-type *Arabidopsis*, 1.34-fold in *atatm-2*, ~2.75-fold in *atatr*, 1.2-fold in *atku80* and *atlig4*, ~1.3-fold in *atku70* and *atpol*λ*-1*, and ~1.4-fold in *atxrcc4*, respectively in presence of 50 μM ABA. This corresponded to ABA mediated inhibition of hypocotyl length elongation was ~14.7% and 52% in DSB sensors/signaling mutants in *atatm-2* and *atatr*, respectively than wild-type *Arabidopsis*. In NHEJ mutants, ~4.4% in *atku80*, 11% in *atku70*, 14% in *atlig4*, 10.2% in *atxrcc4* and 16% in *atpol*λ*-1* reduction of hypocotyl growth were observed than wild-type *Arabidopsis* at 0.5 μM ABA (*P*<0.05–0.01). These results have indicated clear inhibition of hypocotyl length elongation for the DSB sensor/signaling mutant *atatr*.

Again, in contrast to wild-type *Arabidopsis* seedling, which showed ~1.2-fold inhibition in hypocotyl length, the HR-related mutants, including *atmre11*, *atrad50*, *atnbs1*, *atrad51*, *atrad52*, *atrad54 and atbrca1* displayed ~2.25-fold, ~1.77, ~1.8, ~1.7, ~2.0, ~1.9 and ~2.5-fold inhibition of hypocotyl length inhibition due to ABA treatment (0.5 μM ABA) (*p* <0.008–0.01) than untreated control seedlings (Fig 2B). Together, these results have indicated ~31.3%, 26%, 23%, 41.5%, 35.3%, 31.3% and 52% inhibition in hypocotyl length elongation in *atmre11*, *atrad50*, *atnbs1*, *atrad51*, *atrad52*, *atrad54* and *atbrca1*, respectively than wild-type seedlings in presence of higher concentration of ABA (50 μM) (*P*<0.01–0.03). Taken together, these results have revealed considerable level of inhibition of hypocotyl growth in *atatr* and the HR mutants than wild-type *Arabidopsis* and ‘classic’ NHEJ mutants in presence of higher ABA concentration (50 μM ABA).

**Fig 3:**
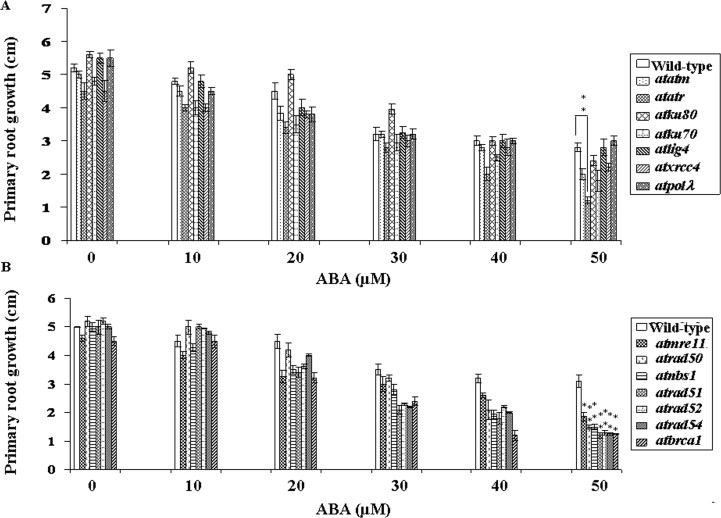
ABA sensitivity of DSB-related mutant seedlings. (A) and (B) Measurement of primary root length in wild-type and DSB-related mutants grown in the absence or presence of increasing concentrations of ABA. 7-days-old pre-germinated wild-type (Wt-Col) and DSB-related mutant seedlings were transferred to MS medium containing different concentrations of ABA and maintained under 16 h light/8 h dark photoperiod for another 7-days before analyzing the relative ABA sensitivity of the seedlings. Bars represent mean values from three independent trials. At least 75 seedlings were counted in each time. *P < 0.05, **P < 0.01 relative to respective controls (n = 3).

Primary root growth of seedlings was also appreciably affected by ABA treatment. As compared to untreated control, whereas wild-type *Arabidopsis* seedlings showed ~1.8-fold inhibition in primary root growth in presence of 50 μM ABA, about 2.0-fold and ~3.75-fold inhibition were detected in *atatm-2* and *atatr* mutants. Among the NHEJ mutants, primary root growth inhibition were ~2.3-fold in *atku80*, ~2.6 in *ku70*, ~1.96 in *atlig4*, ~2.0 in *atxrcc4* and ~1.6-fold in *atpol*λ*-1* as compared to the untreated controls of the respective genotypes (*P<*0.02–0.1) (Fig 3A). Again, in contrast to primary root growth in ABA treated (50 μM ABA) wild-type seedlings, these results corresponded to ~28% and 57% primary root growth inhibition in DSB sensors/signaling mutants (*atatm-2* and *atatr*, *P*<0.01–0.05), while ~14%, 35% and 19.5% inhibition of primary root growth were found in *atku80*, *atku70* and *atxrcc4*, respectively. The primary root growth in *atlig4* and *atpol*λ*-1* did not display any significant difference with the wild-type seedlings under similar condition (Fig 3A).

The HR-related mutants, including *atmre11*, *atrad50*, *atnbs1*, *atrad51*, *atrad52*, *atrad54* and *atbrca1* showed ~2.5, 3.4, 3.3, 4.1, 4.0, 3.9 and 3.6-fold reduction in primary root growth in presence of 50 μM ABA than the untreated control seedlings of the corresponding genotypes (*P<*0.005–0.03) (Fig 3B). These results have indicated ~40%, 51%, 61%, 58% and 59% reduction in primary root growth in *atmre11*, *artad50* and *atnbs1*, *atrad51*, *atrad52*, *atrad54* and *atbrca1*, respectively than wild-type seedlings in presence of 50 μM ABA, suggesting higher sensitivity of HR mutants to exogenous ABA treatment (*P*<0.008–0.01).

**Fig 4:**
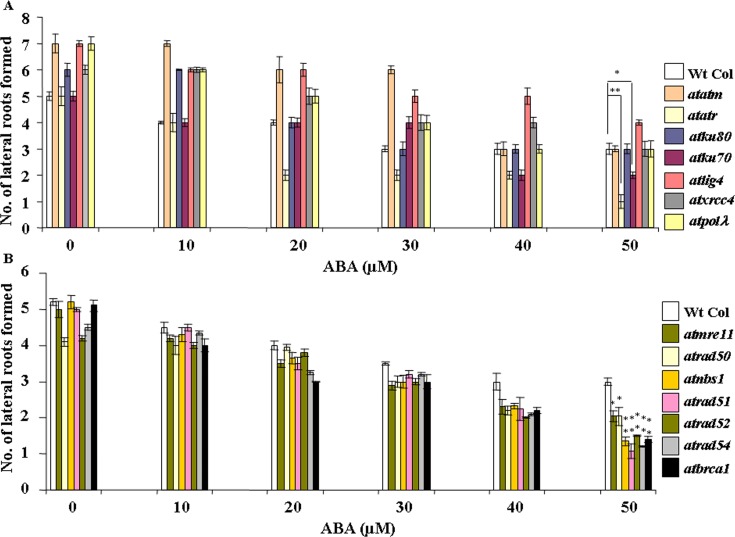
ABA sensitivity and relative levels of lateral root formation in wild-type *Arabidopsis* and DSB-related mutant seedlings. (A) and (B) Comparative analysis of number of lateral root formation in wild-type (Wt-Col) and DSB mutant seedlings in absence and presence of different concentrations of ABA. 7-days-old pre-germinated seedlings were transferred to MS medium supplemented with increasing concentrations of ABA as described under ‘Material and Methods’. Data shown are means ± SD of three independent replications. *P <0.05, *P <0.01 relative to respective controls (n = 3).

Further root growth response studies under ABA stress have also revealed lesser number of lateral root formation in wild-type and DSB-related mutants after exposure to increasing concentrations of ABA (Fig 4). Whereas wild-type (Col-0) seedlings showed ~1.6-fold lees lateral root emergence in presence of 50 μM ABA than untreated control seedlings, ~2.0 and 5.0-fold less lateral root formation was detected in *atatm-2* and *atatr* mutants than the corresponding untreated control seedlings, clearly indicating greater ABA sensitivity of *atatr*. On the other hand, the NHEJ-related mutants showed ~2.0–3.0-fold less lateral root formation (~2.0-fold less in *atku80*, 2.5-fold in *ku70*, 1.75-fold in *atlig4*, 2.0-fold in *atxrcc4* and ~2.3-fold later root emergence in *atpol*λ*-1*, respectively) as compared to the untreated control seedlings of the respective genotypes. Again, these results have indicated ~66.0% and 32.0% less lateral root formation in *atatr* and *atku70*, respectively, than wild-type *Arabidopsis* in presence of 50 μM ABA. The *atatm*, *atku80*, *atlig4*, *atxrcc4* and *atpol*λ showed no significant difference with wild-type *Arabidopsis* in terms of lateral root formation under similar condition (50 μM ABA). Together, these results have indicated notably higher ABA sensitivity of *Arabidopsis atr* mutant than *atm-2* mutant. In addition, *atku70* also displayed greater ABA sensitivity than *atku80* and other components of the ‘classic’ NHEJ pathway.

In HR-related mutants, approximately ~2.4-fold, 2.0, 3.0, 4.6, 2.8, 3.7 and 3.6-fold less lateral root formation was observed in *atmre11*, *atrad50*, *atnbs1*, *atrad51*, *atrad52*, *atrad54* and *atbrca1*, respectively at 50 μM ABA than the untreated control seedlings (Fig 4B) (*P*<0.001–0.03). Together, these results corresponded to ~32%-60% reduction in less lateral root emergence in HR-related mutants than wild-type seedlings in presence of 50 μM ABA (*P*<0.01–0.03).

**Fig 5:**
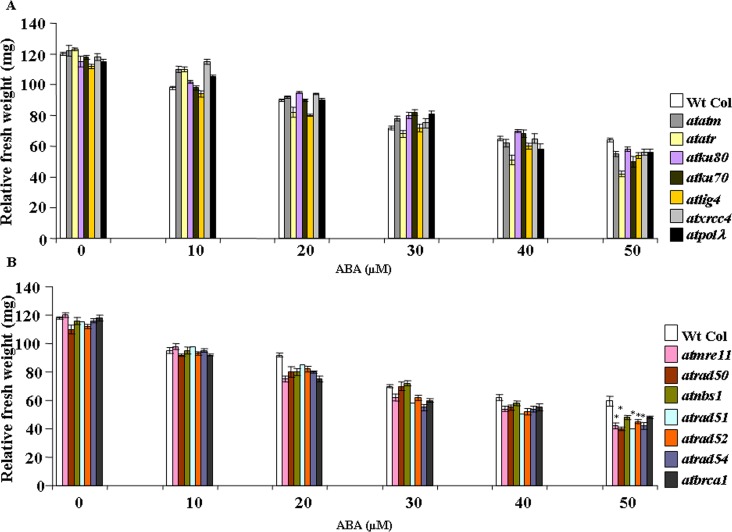
Comparative analysis of fresh-weights of wild-type and DSB-related mutant seedlings under ABA stress. (A) and (B) Determination of relative fresh weights of wild-type and DSB-related mutants grown in the absence or presence of increasing concentrations of ABA. 7-days-old pre-germinated wild-type (Wt-Col) and DSB-related mutant seedlings were transferred to MS medium containing different concentrations of ABA and maintained under 16 h light/8 h dark photoperiod for another 7-days before analyzing the relative ABA sensitivity of the seedlings. Bars represent mean values from three independent trials. At least 75 seedlings were counted in each time. *P < 0.05, **P < 0.01 relative to respective controls (n = 3).

Seedling fresh weights were considerably affected in presence of higher concentrations of ABA in both wild-type and mutant seedlings (Fig 5). About 45% reduction in fresh weight was observed for wild-type *Arabidopsis* seedlings in presence of 50 μM ABA than untreated control condition, while *atatm-2* and *atatr* mutants showed ~54% and 65% decline in seedling fresh weights than the untreated control seedlings of the respective genotypes (corresponding to ~14.0% and 34% reduction in seedling fresh weights than wild-type *Arabidopsis* at 50 μM ABA), indicating greater ABA sensitivity of *Arabidopsis atr* mutant than *atm-2* and wild-type *Arabidopsis*. As compared with the untreated control, the reduction in fresh weights in NHEJ mutant seedlings were ~49%, 57%, 51%, 52% and 51%, as detected for *atku80*, *atku70*, *atlig4*, *atxrcc4* and *atpol*λ*-1*, respectively due to 50 μM ABA treatment (*P*<0.002–0.3). Comparison of fresh weights of wild-type *Arabidopsis* and NHEJ mutants in presence of 50 μM ABA have indicated ~10%, 21%, 15% and 12.5% reduction in fresh weights in *atku80*, *atku70*, *atlig4*, *atxrcc4* and *atpol*λ*-1*, respectively than wild-type seedlings (Fig 5A).

The HR-related mutants showed relatively higher sensitivity in terms of seedling fresh weight reduction due to ABA treatment (50 μM ABA) than the untreated control seedlings. The *Arabidopsis* HR mutants like *mre11*, *rad50*, *nbs1*, *rad51*, *rad52*, *rad54* and *brca1* showed ~65%, 63%, 58%, 65%, 59%, 63% and 59% fresh weight loss during the early seedling stage than the untreated controls (*P*<0.001–0.01) (Fig 5B). This was in contrast with ~49% reduction in wild-type *Arabidopsis* seedling fresh weight in presence of 50 μM ABA than the untreated control seedlings. Furthermore, as compared with 50 μM ABA treated wild-type *Arabidopsis* seedlings, these results corresponded to ~30%, 33%, 20%, 34%, 25%, 30% and 20% reduction in fresh weights in *atmre11*, *atrad50*, *atnbs1*, *atrad51*, *atrad52*, *atrad54* and *atbrca1*, respectively (*P*<0.03–0.05). Together, these results have indicated that the HR mutants are relatively more sensitive to ABA than the NHEJ mutants and wild-type *Arabidopsis*. In addition, the comparison of phenotypic responses of the wild-type and DSB-related mutants using the seedling growth related morphological parameters in absence or presence of exogenously applied higher concentrations of ABA during germination and early stages of seedling growth have revealed prominent ABA mediated growth inhibition due to DNA damage during seed germination and post-germination seedling growth, suggesting role of ABA in the induction of DNA damage and genome instability. However, it was also interesting to note the lack of clear correlation between ABA sensitivity of the mutants in terms of the morphological response and the relative reduction in fresh weights of the seedlings under ABA stress. For the DSB mutants, although seed germination rate, hypocotyl and primary root length and number of lateral root emergence decreased notably due to ABA treatment, overall growth abnormalities, such as thickening and curling of primary leaves, hypocotyl etc. did not reveal the actual reduction in growth rate in terms of seedling fresh weights. This effect was particularly prominent in HR mutants where inhibition in seedling growth was not clearly reflected in terms of reduction in seedling fresh weights following ABA treatment.

### ABA treatment generates DNA strand breaks

**Fig 6:**
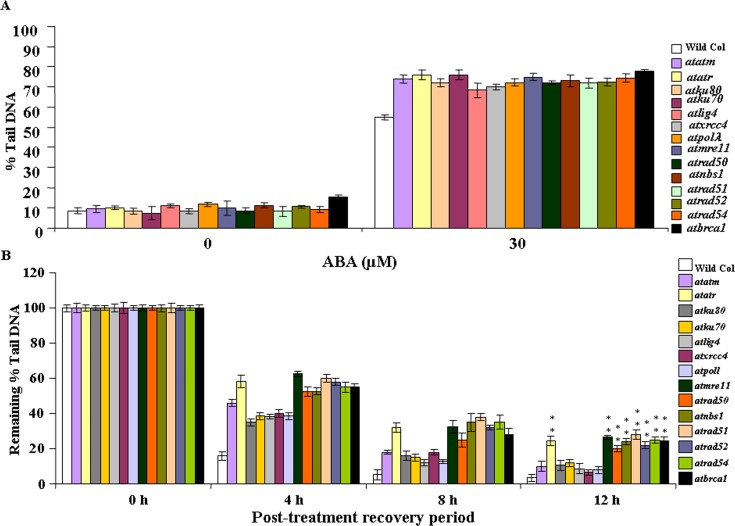
ABA-induced DNA breaks and repair activity in wild-type and DSB-related mutants. (A) Two-week-old wild-type and DSB-related mutant seedlings were treated with 30 μM ABA for 24 h at room temperature and the extent of DSB induction was analysed by the neutral (N/N) comet assays. The extent of DNA damage is represented by the fraction of DNA migrating in the comet tail. (B) DSB repair during various post-treatment recovery periods, including 0 h, 4 h, 8 h and 12 h, respectively. Additional data sets for 2 h and 6 h of post-treatment recovery periods are available on request. Mean percentage of DNA in the comet tails of wild-type and other mutant lines at various recovery time points under standard growth conditions. Maximum damage is normalized as 100% at t = 0 for all lines. Data shown are means (±SD) of three independent replications.

Previous studies have indicated that ABA or ABA signaling pathway affects DNA replication machinery in plants, induces DNA strand breaks and causes genome instability [5,43]. Unrepaired or error-prone repair of DNA damage, particularly DNA strand breaks, in actively dividing plant tissues considerably affect plant growth by inhibiting DNA replication and transcription, finally disruption of cell cycle progression. Therefore, it was next addressed whether the growth inhibition and sensitivity of wild-type *Arabidopsis* and other DSB-related mutants is at least partly due to DNA damage and associated impairment of repair activity. Comet assay was carried out under neutral conditions [29] using nuclear suspension prepared from the untreated control and 30 μM ABA treated two-week-old wild-type and DSB-related mutant seedlings to measure the relative level of accumulation of DSBs and the repair rates (Fig 6). In general, the DSB-related mutants including the NHEJ and HR-related mutants showed approximately ~15–20% higher DSBs than wild-type seedlings due to 30 μM ABA treatment for 24 h (Fig 6A). In particular, *atatm-2* and *atatr* mutants showed more than 25% higher DSB accumulation than wild-type *Arabidopsis* following ABA treatment (30 μM). The NHEJ mutants, including *atku80*, *atlig4*, *atxrcc4* and *atpol*λ*-1* were found to accumulate ~20% higher DSBs than wild-type *Arabidopsis* under ABA stress. DSB accumulation was relatively higher in *atku70* than other NHEJ mutants. On the other hand, the HR mutants showed ~24–30% higher DSB accumulation than wild-type Arabidopsis due to ABA treatment.

The DSB repair kinetics study was then carried out to investigate the relative DSB repair activities in wild-type and DSB-related mutants. After 30 μM ABA treatment for 24 h, seedlings were transferred to one-half strength of liquid MS-medium and recovered for up to 12 h. In wild-type seedlings, whereas approximately 90–98% DSB repair activity could be detected during 6–12 h of recovery, the NHEJ-related mutants and *atatm-2* showed approximately 75–90% repair activity under similar recovery conditions (Fig 6B). The *atatr* mutant line showed ~60–75% DSB repair during the later recovery periods (6–12 h post-treatment) (*P*<0.008–0.001) (Fig 6B). As compared with wild-type and NHEJ-related mutants, the DSB repair activity was appreciably less in HR mutants during the initial recovery periods (2–4 h) and this was maintained till the late recovery phases. In wild-type seedlings, whereas more than 95% DSB repair activity was detected after 12 h of treatment, the HR-related mutants, including *atmre11*, *atrad50*, *atnbs1*, *atrad52*, *atrad54* and *atbrca1* showed ~72–78% recovery under similar condition (*P*<0.001–0.02). Interestingly, *atrad51* mutant showed relatively better repair activity during the later phases of recovery phases (6–12 h post-treatment) (Fig 6B), however, it was still about 8-fold less recovery than wild-type *Arabidopsis* and 2.5–3.5-fold less than other NHEJ mutants. Together, these results have indicated appreciable difference in HR and NHEJ-related mutants in repairing the ABA-induced DNA breaks during the recovery periods after ABA treatment. The less DSB repair activity in HR-related mutants have further suggested loss-off-function of major HR-related genes probably impairs the repair activity of DNA strand breaks induced due to ABA treatment in *Arabidopsis* seedlings.

### ABA-mediated DNA double-strand breaks activate damage repair signaling via histone H2AX phosphorylation

**Fig 7:**
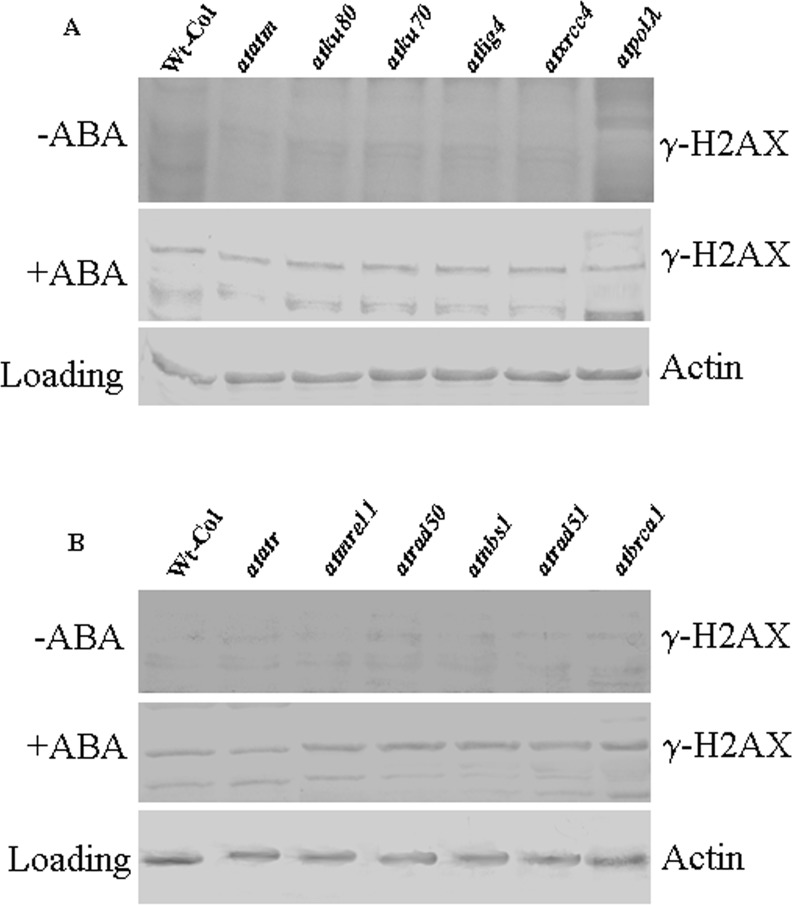
Detection of histone H2AX phosphorylation in wild-type and DSB-related mutants following ABA treatment. (A) and (B) Two-week-old wild-type (Wt-Col), *atatm-2*, *atatr*, the ‘classic’ NHEJ-related mutants and HR mutant seedlings were exposed to 30 μM for 24 h following which histone protein extracts were prepared. Immunodetection of γ-H2AX was carried out using rabbit antiplant γ-H2AX polyclonal antibody in total protein extracts (60 μg). The upper panels in each figure represent Western bolts from untreated control seedlings while the lower panels indicate expression levels of actin protein, detected using anti-actin antibody, as a loading control. Figures are representative blots from two experiments.

One of the well known immediate responses to the induction of DSBs includes rapid phosphorylation of large numbers of the histone variant H2AX precisely at the site of DNA damage [44]. Phosphorylated H2AX, recognized as γ-H2AX, has been shown to form ‘foci’ at the site of DSBs generated in response to genotoxic agents, such as ionizing radiation [45], replication stress [46] and recombination during meiosis [47]. In plants, although many of the signaling components operating downstream of the ATM and ATR kinases have not yet been well characterized, the ATM-dependent phosphorylation of H2AX, which actually serves as a highly conserved signal for the induction of DSBs, occurs rapidly following generation of DSBs [48]. Whereas an ATM-dependent H2AX phosphorylation has been observed following induction of DSBs by genotoxins like X-rays and bleomycin [49], H2AX phosphorylation via ATR generally occurs during the S-phase of cell cycle due to replication block [43]. In our study, ABA treatment of wild-type (Col-0) and DSB-related mutants caused the induction of H2AX phosphorylation as detected in immunoblotting experiments with equal amount of protein extracts [50] using the plant specific H2AX antibody, which recognized a 16-kDa protein in the extracts prepared from ABA treated wild-type and DSB-related mutant seedlings of *Arabidopsis* (Fig 7). The antibody also recognized very low level of γ-H2AX protein in extracts from untreated control seedlings (Fig 7A and 7B, upper panel), which probably indicated accumulation of marginal level of phosphorylated H2AX. Cross-reactivity of the polyclonal antibody with the non-phosphorylated protein may also be another factor as reasoned in earlier studies [32]. In addition to wild-type, NHEJ and HR-related mutants, the plant γ-H2AX antibody detected the 16-kDa protein in ABA treated extracts from both *atatm-2* and *atatr* mutant lines (Fig 7A and 7B, lane 2), indicating possible functional involvement of ATM kinase in *atatr* and ATR kinases in *atatm-2* mutant, respectively, in the phosphorylation of H2AX following DSB induction due to ABA treatment. Strong signal for histone H2AX phosphorylation was also detected in immunoblotting experiments using protein extracts from ABA treated *Arabidopsis* seedlings of two other core HR pathway gene mutants, including *atrad52* and *atrad54*, respectively (data not shown). Furthermore, as compared with wild-type and ‘classic’ NHEJ-related mutants, our preliminary results have detected considerable level of phosphorylated H2AX in the protein extracts from HR-related mutants even during the later phases of recovery after ABA treatment (data not shown). Together these results establish the link between ABA-mediated induction of DSBs in plant genome and activation of DNA damage response via H2AX phosphorylation by ATM and ATR kinases. The detection of phosphorylated H2AX during the post-treatment recovery periods in HR-related mutants also couples functional involvement of HR genes in repair of ABA-induced DSBs. However, this observation needs further extensive analysis.

### ABA treatment induces the expression of HR-related genes in *Arabidopsis* seedlings

Our results on germination response and post-germination growth of *Arabidopsis* seedlings using wild-type and DSB-related knockout mutant lines revealed relatively higher ABA sensitivity in HR mutants (Figs 1–5). In addition, analysis of DSB repair kinetics in ABA treated *Arabidopsis* seedlings indicated clearly delayed rate of DSB repair in HR-related mutants (Fig 6), suggesting possible function of HR genes in repairing DSBs, generated due to ABA treatment in *Arabidopsis* during early stages of seedling growth.

**Fig 8:**
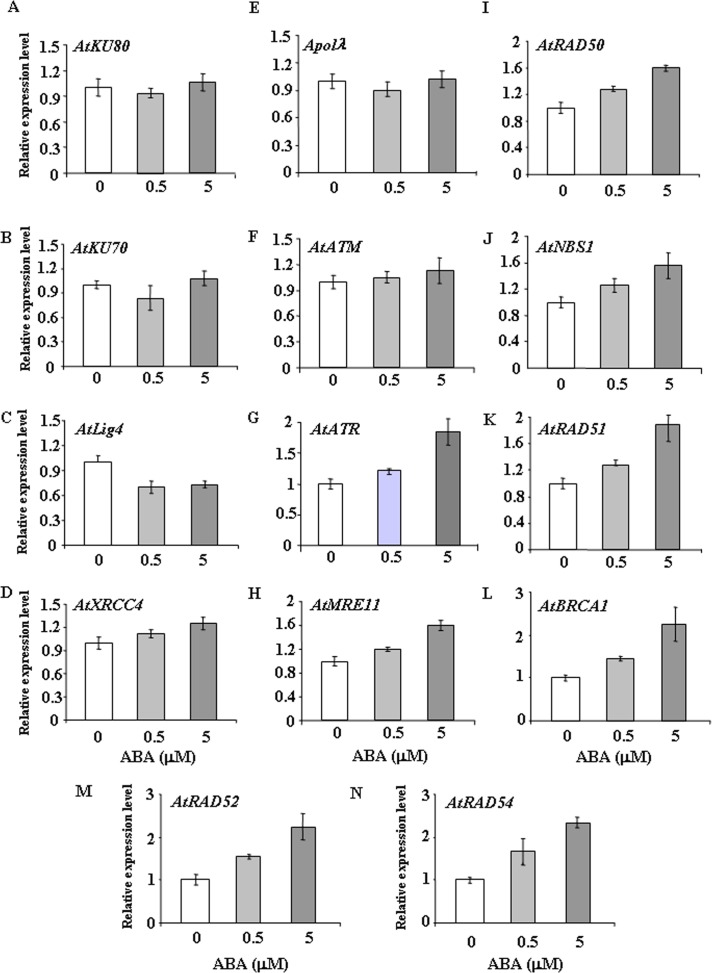
ABA treatment induces expression of DSB-related HR genes during seed germination. Relative expression levels of the major DSB-related marker genes during seed germination in wild-type *Arabidopsis* in the absence or presence of 0.5 and 5 μM ABA. The transcripts of each gene under normal condition were used a comparison standard. The expression levels were determined by real-time qPCR and normalized to *ACTIN2* (*ACT2*). The data are representative from one of three independent trials with similar results. Error bars indicate ±SE (n = 3).

To further determine the possible involvement of HR genes in repair of ABA induced DSBs, we next analysed the steady-state transcript levels of major DSB-related genes in wild-type *Arabidopsis* during seed germination in presence of 0.5 and 5 μM ABA (Fig 8). ABA treatment during seed germination did not considerably influence the transcript levels of the ‘classic’ NHEJ genes, including *AtKU80*, *AtKU70*, *AtLig4*, *AtXRCC4* and *AtPol*λ, respectively. Whereas *AtKU80*, *AtKU70* and *AtPol*λ transcript levels remained almost unaffected at 0.5 and 5 μM ABA, *Atlig4* expression was repressed under similar condition. *AtXRCC4* transcript level increased only marginally at 5 μM ABA (~1.1–1.2-fold increase) (Fig 8A–8E). As compared with untreated control, endogenous message levels of *AtATR* increased in presence of 5 μM ABA (~1.2-2-fold induction at 0.5–5 μM ABA), while marginal induction could be detected for *AtATM* (0.8–1.1-fold induction in presence of 0.5–5 μM ABA) (Fig 8F and 8G). Clear change in the expression pattern of HR genes was observed. An increase in *AtMRE11*, *AtRAD50*, and *AtNBS1* (~1.2–1.6-fold) mRNA levels were detected in presence of 0.5–5 μM ABA in germinating seeds of wild-type *Arabidopsis*. *AtRAD51* transcript level increased ~1.2-fold at 0.5 μM ABA, while almost 2-fold induction was detected at 5 μM ABA (Fig 8H–8K). For other core HR genes, including *AtBRCA1*, *AtRAD52* and *AtRAD54*, clear change in the expression pattern was detected due to ABA treatment. *AtBRCA1* expression level increased approximately 1.4–2.5-fold in presence of 0.5–5 μM ABA (Fig 8L). On the other hand, ~1.5–2.5-fold induction of *AtRAD52* and *AtRAD54* transcript level could be detected in presence of 0.5–5 μM ABA (Fig 8M and 8N). Together, these results have revealed transcriptional up-regulation of the core HR pathway genes during seed germination in presence of exogenously applied ABA.

**Fig 9:**
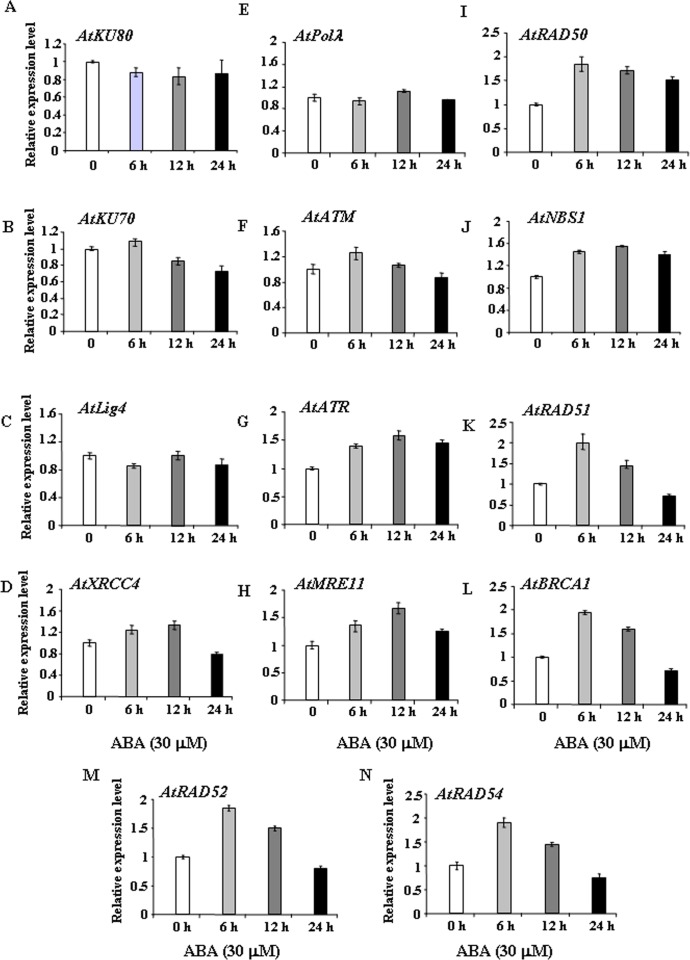
ABA treatment increases expression of HR genes during post-germination periods. Two-week-old wild-type *Arabidopsis* seedlings were treated with 30 μM ABA for the indicated time points. The expression levels of the major DSB-related marker genes were determined by real-time qPCR and normalized to *ACTIN2* (*ACT2*). The data are from one experiment with triple technical repeats. Error bars indicate ±SE, n = 3.

To further validate the these results, 7-days-old wild-type *Arabidopsis* seedlings were treated with 30 μM ABA for various time points and then transcript abundance of the selected DSB related genes were analyzed. Detectable level of changes, if any, in the transcript abundance of the DSB repair genes were almost negligible during the early time points (1–3 h) (data not shown). Any appreciable changes in the expression levels of *AtKU80*, *AtLig4* and *AtPol*λ could not be detected further (Fig 9A, 9C and 9E). ABA treatment down-regulated *AtKU70* expression at the later time points while only marginally increased *AtXRCC4* expression during 6–12 h of treatment, followed by a decline at the later stage (Fig 9B and 9D). An initial marginal increase in *AtATM* mRNA level was observed after 6 h of treatment and then it decreased gradually at the later time points. In contrast, *AtATR* transcript level increased more than 1.5-fold than control during the 6–12 h period (Fig 9F and 9G). During 6–12 h of ABA treatment, *AtMRE11*, *AtRAD50* and *AtNBS1* transcript levels increased more than about 1.2–1.6-fold over the control (Fig 9H–9J), while *AtRAD51*, *AtBRCA1*, *AtRAD52* and *AtRAD54* mRNA levels showed about 2-fold increase during the early period (6 h) of treatment. However, the transcript levels then declined at the later stages (Fig 9K–9N). The increased transcript abundance of the core HR genes corroborated well the with the previous observations of greater ABA sensitivity of the HR mutants, suggesting possible functional involvement of HR mediated DSB repair pathway in *Arabidopsis* after ABA treatment for repairing the ABA induced DSBs in the genome [5].

## Discussion

Various phytohormones are involved in regulating the stress responses in plants by acting as key modulator of cell division via controlling the expression of cell-cycle regulated genes [51]. Abscisic acid (ABA), produced under abiotic stress conditions, plays pivotal role in plant stress response [52]. Earlier studies have demonstrated ABA-mediated inhibition of cell-division through increased activity of a cyclin-dependent protein kinase inhibitor KRP1/ICK1 [53]. In addition, DNA synthesis and cell cycle related proteins are directly targeted and inhibited by ABA or ABA signaling, resulting in inhibition of DNA synthesis and cell cycle arrest [54]. In this study, we have further investigated the influence of ABA on DNA damage repair machinery in plant genome. Analysis of germination frequency and post-germination growth responses of wild-type and DSB related knockout mutants in presence of various concentrations of ABA have revealed higher ABA sensitivity of HR-related mutants, indicating functional association of DSB repair pathway with ABA mediated genome instability. Comet analysis has indicated slower DSB repair activity in HR-related mutants subjected to ABA treatment. Increased transcript abundance of HR-related genes in wild-type *Arabidopsis* during seed germination and early stages of seedling growth under ABA stress have provided further evidence to suggest involvement of HR pathway in repairing DNA strand breaks induced by ABA treatment in *Arabidopsis*.

Understanding the interaction between DNA damage sensing, repair and hormone signaling is far more advanced in animal system. However, plethoras of evidences have indicated that abiotic stress induced accumulation of ABA may influence genome stability through interactions with the components of HR and NHEJ as well as other repair pathways [55]. Various abiotic stress factors, including high salinity, water deficit and temperature stress promote the accumulation of ABA in plant cell, leading to the induction of several ABA responsive genes involved in the regulation of ABA linked stress responses [56]. Therefore, understanding the possible link between ABA signaling and genotoxic stress mediated genome instability has become one of the emerging area of study during the past couple of years in the model system *Arabidopsis* and other crop plants.

As like other organisms, although transcriptional changes are considered as an integral part of DNA damage response in plants, information is still limited about the influence of plant stress hormones like ABA on the transcriptional regulation of repair genes involved in repairing DNA strand breaks. In pea, exposure to abiotic stress including ABA have been shown to enhance the expression of topoisomerse 2 (TOP 2), an essential component of DNA replication machinery in plants [57]. Earlier studies in *Arabidopsis* involving characterization of an ABA overly sensitive mutant *abo4-1*, with impaired DNA Pol ε function [5], has provided important clue regarding interaction between DNA repair pathway and ABA signaling. ABA treatment has been shown to enhance the HR frequency in both wild-type and *abo4-1* mutant. The ABA overly sensitive mutant, *abo4-1* in *Arabidopsis*, produced due to mutation of *POL2a/TILTED1* (*TIL1*) gene, encoding the catalytic subunit of DNA polymerase ε (Pol ε), showed increased sensitivity to ABA. In *abo4/pol*ε mutant, ABA treatment caused DNA double-strand breaks and genome instability with increased expression of some DSB-inducible genes like *GR1 and MRE11* and high rate of homologous recombination [5]. In *abo4-1* mutant, ABA treatment caused up-regulated expression of DSB sensor *MRE11* and other HR genes, such as *RAD51* and *BRCA1*, while repressed expression of ‘classic’ NHEJ genes like *Ku70*. Since treatment with genotoxic agent MMS increased the expression of all these genes, it was suggested that DNA damage response induced by ABA and MMS operates via distinct pathways. Furthermore, the induction of ABA mediated DSBs in *abo4-1* mutant was suggested to be repaired via the HR pathway with the involvement of ABA-mediated up-regulation of *MRE11*, since ABA repressed *Ku70* expression, thus, reducing ‘classic’ NHEJ activity [5]. Subsequent research has demonstrated role of ABA in reactivation of the transposon *TS1* in *abo4/pol*ε, enhancing genome instability [43]. More recent studies have indicated that ABA regulates the function of replication factor C1 (RFC1), the large subunit of RFC complex required for loading proliferating cell nuclear antigen (PCNA), in somatic homologous recombination in *Arabidopsis*. As like *abo4/pol*ε, *rfc1* mutant showed enhanced sensitivity to ABA in terms of seed germination and root growth. However, in contrast to *abo4/pol*ε, ABA treatment compromised the HR rate and expression of major DNA damage responsive genes in *rfc1*, suggesting role of RFC1 in ABA mediated HR in *Arabidopsis* [43]. In our study, in contrast to the ‘classic’ NHEJ genes, such as *KU80*, *KU70*, *Lig4*, *XRCC4* and *Pol*λ, exogenous ABA treatment increased the expression of most of the HR-related genes, such as *MRE11*, *RAD50*, *NBS1*, *RAD51* and *BRCA1* during seed germination and particularly during early stages of seedling growth in wild-type *Arabidopsis* (Figs 8 and 9). Up-regulation of *AtATR* was expected since ABA treatment is known to inhibit replication possibly via ROS generation and oxidative stress. However, due to ABA treatment, slightly more than 1.2-fold induction was detected for *AtATM* mRNA in both germinating *Arabidopsis* seeds and seedlings, indicating activation of ATM kinase activity under replication stress, which may also generate DSBs due to inefficient repair of collapsed replication forks.

As initially demonstrated in mammals, the multifunctional protein complex, comprising of three proteins, such as MRE11, RAD50 and NBS1, commonly known as the MRN complex, plays crucial role in the detection of DSBs [58]. A key function of the MRN complex is associated with the C-terminal domain of NBS1 protein, which recruits the protein kinase ataxia telangiectasia mutated (ATM) to the site of DSBs, therefore activating the ATM kinase function, initiating the signaling cascade [59]. However, besides role in DNA damage detection and signaling, the MRN complex is also involved in both of the major types of DSB repair pathways, including NHEJ and homologous recombination (HR). Furthermore, the functional involvement of MRN complex in damage detection and signaling via activation of ATM-kinase activity has been shown to be conserved in plants [60] and the initial mechanism of DSB detection has also been shown to influence the choice of repair pathway. The involvement of components of MRN complex has also been implicated in ‘non-classical’ NHEJ such as micro-homology mediated NHEJ pathway [36]. However, previous studies have indicated that in contrast to the KU80-KU70 heterodimer, which function in DSB detection mainly in NHEJ mediated repair, the components of MRN complex play important function in DSB detection and signaling in HR mediated repair [37]. In our study, ABA sensitivity of the *Arabidopsis* mutants of subunits of MRN complex and the mutants of other core components of HR pathway, such as RAD51, RAD52, RAD54 and BRCA1 provided evidence for the involvement of HR mediated DSB repair pathway following ABA treatment.

As mentioned before, the HR pathway operates mainly via the activity of RAD52 epistasis groups of proteins, which include RAD51, RAD52, RAD54, RAD55, RAD57 and the MRN complex, comprising of MRE11, RAD50 and NBS1, respectively [36]. Another most important feature of the HR pathway is the DSB repair based on homologous sequence and thus intimately associated with the activity of RAD51, which is involved in the search of homologous sequence. The RAD51 mutants in *Arabidopsis* showed considerable sterility, indicating important function of RAD51 in DNA repair [61]. Evidence from maize has demonstrated roles during seed germination, where RAD51-deficient seeds produced abnormal seedlings following genotoxin treatment [62], establishing the key role of RAD51 in HR pathway. As found in our study, compared with the HR mutants, the NHEJ mutants showed mild sensitivity to ABA treatment. On the other hand, Arabidopsis mutants of the core HR pathway genes, including *rad51*, *rad52*, *rad54* and also *brca1* showed greater ABA sensitivity in terms of germination response and ABA mediated growth inhibition during the early stages of seedling development. In addition, approximately 1.8–2.0-fold induction of *RAD51*, *RAD52*, *RAD54* and *BRCA1* transcripts could be detected in wild-type *Arabidopsis* during germination and early seedling stages under ABA stress, suggesting involvement of HR mediated repair of ABA-induced DNA damage.

Our results have also indicated the ABA sensitive phenotype of *atbrca1*, as observed during the seed germination and early seedling development stage. Previous studies have demonstrated role of BRCA1 in homologous recombination and DNA damage signaling with up-regulated expression in response to DNA damage [53]. Involvement of BRCA1 in HR mediated DSB repair has been indicated in previous research as BRCA1-deficient plants showed impaired HR activity [63]. The BRCT domain, present in BRCA1 and many other proteins functioning in DNA damage response and cell cycle check points, regulates protein-protein interactions in mammals and plants. BRCA1 has also been shown to regulate the choice of DNA double-strand-break repair pathway throughout the cell cycle [64]. Notable induction of *AtBRCA1* transcript in wild-type *Arabidopsis* during seed germination and post-germination phases along with the delayed rate of DSB repair in *atbrca1* mutant following ABA treatment indicated possible role of BRCA1 in repair of DSBs generated under ABA stress. However, additional research is required to further characterize the functional involvement of BRCA1 and its interacting network in HR mediated repair of DSBs generated by ABA probably via ROS production and oxidative stress.

In plants, DSB detection is mainly carried out by the complex of KU70 and KU80 [21]. The Ku genes, encoding the Ku heterodimer protein, play crucial role in DNA repair, regulation of cell cycle control and telomere maintenance. Characterization of *Arabidopsis ku* mutants and their phytohormone mediated regulation have suggested important cross-talk between DNA repair signaling and phytohormone networks. In eukaryotes, the NHEJ pathway involves various protein factors and studies in mammals, yeast and plants have shown the involvement of some conserved core components, including Ku70-Ku80 complex, DNA polymerase λ and the DNA ligase 4-XRCC4 complex [23,36]. The Ku70-Ku80 complex shows high affinity for broken DNA ends and protects exposed DNA ends from exonuclease mediated degradation [65]. *Arabidopsis ku* mutants have been found to display greater dependence on micro-homology mediated DSB repair. *Arabidopsis ku70* mutants display longer telomere than wild-type, while *ku80* mutants have normal telomerase activity but still with longer telomere, indicating important role of Ku70 in telomere maintenance [66,67].

Earlier study has indicated modulation of Ku activity via phytohormone mediated pathway [68]. ABA has been found to repress the expression of both *Ku80* and *Ku70* following heat stress, which causes higher accumulation of ABA in plants [69]. In another study, UV-sensitive *Arabidopsis* mutant *uvs66* was shown to be hypersensitive to salinity and ABA. Under salinity stress, which promotes ABA accumulation in plants, increased abundance of the major HR gene, *RAD51* was detected in *uvs66* mutant background. On the other hand, despite hypersensitivity to ABA, *uvs66* mutants did not show any significant change in *RAD51* expression following ABA treatment [70]. Together, these observations have suggested complex interactions of ABA-dependent and ABA-independent stress signaling with DNA damage response and signaling.

In our study relatively higher ABA sensitivity of *ku70* mutant than other ‘classic’ NHEJ mutant was noted. Relatively higher ABA sensitivity of *ku70* mutant than the other ‘classic’ NHEJ mutants was puzzling as we expected similar level of sensitivity like other NHEJ mutants. However, as mentioned above, in *Arabidopsis*, studies involving both *ku70* and *ku80* mutants have shown longer telomere under both mutant backgrounds. While *ku70* showed aberrant telomerase activity it was normal in ku80 [66,67], suggesting greater contribution of Ku70 in telomere maintenance. This observation probably also indicate the differential affinity of Ku70 and Ku80 proteins for broken DNA ends. Furthermore, scanning of up to ~2000 bp (~2 kb) upstream genomic sequence of Arabidopsis thaliana Ku80 and Ku70 genes have revealed overrepresentation of ABA responsive elements (ABRE) and ABRE-like *cis*-regulatory elements (TACACGTA, CACGT) in the promoter of Ku70 (at least 6 ABRE or ABRE-like motifs) as compared with Ku80 which showed fewer ABREs (2 ABRE or ABRE-related motifs). This appears to be one possible reason for higher ABA sensitivity of Ku70 than Ku80. However, further experimental verification is required.

HR pathway involves high fidelity DNA repair synthesis by utilization of intact homologous sequences as template in the repair of DSBs generated due to inhibition of replication [20]. On the other hand, although it has not been well defined that how ABA directly targets DNA synthesis, ABA mediated production of ROS seems to induce oxidative stress which finally causes replication stress. Prolong replication stress due to collapsed replication fork generates DNA strand breaks and possibly stimulates HR pathway via the activation of ATR kinase, which is induced by replication stress and specifically phosphorylates the downstream targets, including phosphorylation of histone H2AX. Phosphorylation of H2AX was confirmed in immunoblotting experiments using extracts from wild-type *Arabidopsis* and DSB related mutants (Fig 7), suggesting ABA mediated induction of DSBs probably via ROS production and oxidative stress. Furthermore, up-regulation of some major antioxidant genes, including *catalase 1* (AT1G20630), *peroxidase* (AT1G05240) and *superoxide dismutase* (AT1G08830) in wild-type (Col-0) and DSB related mutants under ABA stress during seed germination and early seedling development (data not shown) also supported this idea.

**Fig 10:**
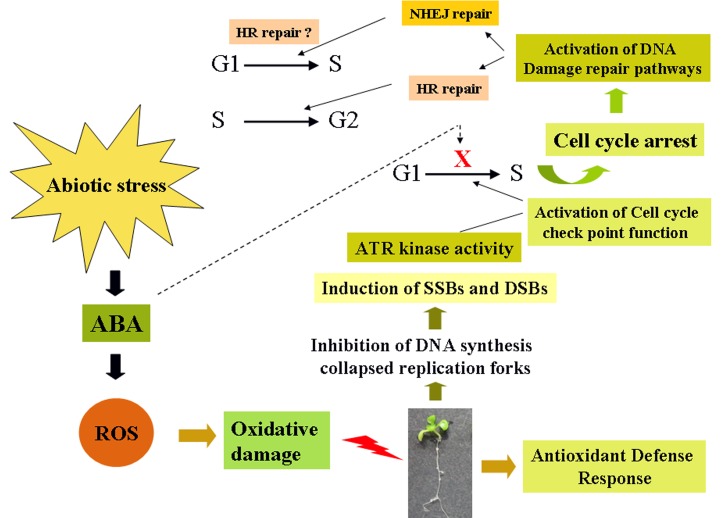
Proposed hypothetical model illustrating the interaction between ABA mediated genome instability and possible involvement of HR pathway. ABA mediated reactive oxygen species (ROS) generation and oxidative damage inhibits DNA replication. Prolong replication block results in the formation of DNA single and double stand breaks, which activate ATR kinases and cell-cycle arrest. Activation of DNA double-strand break repair pathway for repairing damage during G1-S and S-G2 phases of cell-cycle. Possibility of HR repair during G1-S has been indicated with sign of interrogation.

Based on a proposed model, it has been suggested that DSBs generated due to stalled replication fork are repaired via HR when replication fork encounters DNA damage and ceases [5]. ABA phenotype of *atatr* mutant during seed germination and early stages of seedling growth, along with enhanced transcript abundance of *AtATR* in wild-type *Arabidopsis* following ABA treatment supports our notion on ABA mediated inhibition of replication and DNA strand breaks followed by activation of ATR kinase and probable recruitment of HR pathway. Previous studies have indicated ABA-mediated inhibition of DNA replication in synchronized BY-2 cells in tobacco. ABA treatment was found to negatively influence cell cycle progression at the G1/S transition phase while appeared to be less effective on G2/M transition phase in tobacco BY-2 cells and also in germinating maize seeds [24,71]. In addition, previous studies have indicated functional involvement of HR-pathway during the late S-G2 phase of cell cycle for repairing broken DNA strands with high fidelity. In contrast, the NHEJ pathway becomes functional mainly during the G1 to early S phase of cell cycle for repairing DNA strand breaks irrespective of sequence homology as at this point sister chromatids are not available for utilization as homologous template [72]. This implies the question on whether HR genes also function during G1-S transition phase following ABA mediated disruption of replication through oxidative stress, causing activation of ATR kinase and cell-cycle check point functions (Fig 10). Interestingly, ABA treatment did not significantly affect the expression of G2-associated cyclin *CYCB1;1* in wild-type and other DSB related mutants (data not shown), consistent with the previous notion that ABA affects the progression of cell cycle at the G1-S phase [24]. However, our preliminary observations have indicated increased endogenous message levels of *AtATR* and HR genes including *AtBRCA1* under NHEJ mutant background (single mutants of the major ‘classic’ NHEJ genes) along with up-regulated expression of *H4b* histone (S-phase specific *Histone4*) in both NHEJ and HR mutants under ABA stress (unpublished data), suggesting possible function of HR genes in DSB repair following ABA treatment in prolonged S-phase, as observed in earlier studies with increased expression of *H4b* histone, indicating longer S-phase [73,74]. However, further extensive studies at the molecular and genetic level are essential to understand the interaction between ABA mediated genome instability and the functional involvement of DSB repair pathways in plant genome.

## Supporting Information

S1 File Supplementary materials.doc. (DOC)

S1 Table Primer sequences used for PCR genotyping for the characterization of T-DNA insertion mutant lines in *Arabidopsis thaliana*. (DOC)

S2 Table Primer sequences used for transcript profile analysis by quantitative real-time PCR. (DOC)

S1 Fig Schematic structure of major marker genes involved in the detection, signaling and repair of DNA double strand breaks in *Arabidopsis*. (PPT)

S2 Fig Characterization of T-DNA insertion mutant lines for DSB-related genes. (PPT)

## Abbreviations

ABA: abscisic acid
DSBs: double-strand breaks
HR: homologous recombination
NHEJ: non-homologous end joining
Pol: polymerase
qRT-PCR: quantitative real-time PCR
