# Supplementary material for: Homologous Recombination Defective Arabidopsis Mutants Exhibit Enhanced Sensitivity to Abscisic Acid
Source: PLoS One. 2017 Jan 3;12(1):e0169294. doi: 10.1371/journal.pone.0169294 (PMC5207409; doi:10.1371/journal.pone.0169294)
Supplement: S1 File — (DOC) [file pone.0169294.s001.doc]

**Supplementary Materials and methods**

**RNA isolation and transcript profile analysis by Semi quantitative RT-PCR**

Transcript profile was analysed by semi quantitative RT-PCR following the protocol described previously [1]. Total RNA was isolated from ~100 mg of seedling tissues using RNeasy plant minikit (Qiagen) following manufacturer’s instruction. RNA samples were treated with DNase I (RNase free, Roche) according to manufacturer’s protocol to remove genomic DNA contaminations. First strand cDNA was synthesized from 1 μg of total RNA sample using AMV first strand cDNA synthesis kit (Roche) following manufacturer’s instructions. For semi-quantitative RT-PCR analysis for *AtKU80*, a first cycle of 2 min at 94°C, 45 s at 53°C and 1 min at 72°C was followed by 45 s at 94°C, 45 s at 53°C and 1 min at 72 °C for 22 cycles. For *AtKu70*, a first cycle of 2 min at 94°C, 45 s at 53°C and 1 min at 72°C was followed by 45 s at 94°C, 45 s at 53°C and 1 min at 72 °C for 22 cycles. For *AtLig4*, a first cycle of 2 min at 94°C, 45 s at 53°C and 1 min at 72°C was followed by 45 s at 94°C, 45 s at 53°C and 1 min at 72 °C for 22 cycles. For *AtXRCC4*, a first cycle of 2 min at 94°C, 45 s at 55°C and 1 min at 72°C was followed by 45 s at 94°C, 45 s at 55°C and 1 min at 72 °C for 22 cycles. For *AtPolλ* transcripts, a first cycle of 2 min at 94°C, 45 s at 49°C and 1 min at 72°C was followed by 45 s at 94°C, 45 s at 49°C and 1 min at 72 °C for 22 cycles. For *AtATM*, a first cycle of 2 min at 94°C, 45 s at 55°C and 1 min at 72°C was followed by 45 s at 94°C, 45 s at 55°C and 1 min at 72 °C for 22 cycles. For *AtATR*, a first cycle of 2 min at 94°C, 45 s at 55°C and 1 min at 72°C was followed by 45 s at 94°C, 45 s at 55°C and 1 min at 72 °C for 22 cycles. For *AtMRE11*, a first cycle of 2 min at 94°C, 45 s at 53°C and 1 min at 72°C was followed by 45 s at 94°C, 45 s at 53°C and 1 min at 72 °C for 22 cycles. For *AtRAD50*, a first cycle of 2 min at 94°C, 45 s at 55°C and 1 min at 72°C was followed by 45 s at 94°C, 45 s at 55°C and 1 min at 72 °C for 22 cycles. For *AtNBS1*, a first cycle of 2 min at 94°C, 45 s at 53°C and 1 min at 72°C was followed by 45 s at 94°C, 45 s at 53°C and 1 min at 72 °C for 22 cycles. For *AtRAD51*, a first cycle of 2 min at 94°C, 45 s at 47°C and 1 min at 72°C was followed by 45 s at 94°C, 45 s at 47°C and 1 min at 72 °C for 22 cycles. For *AtRAD52*, a first cycle of 2 min at 94°C, 45 s at 55°C and 1 min at 72°C was followed by 45 s at 94°C, 45 s at 55°C and 1 min at 72 °C for 22 cycles. For *AtRAD54*, a first cycle of 2 min at 94°C, 45 s at 55°C and 1 min at 72°C was followed by 45 s at 94°C, 45 s at 55°C and 1 min at 72°C for 22 cycles. For *AtBRCA1*, a first cycle of 2 min at 94°C, 45 s at 53°C and 1 min at 72°C was followed by 45 s at 94°C, 45 s at 53°C and 1 min at 72 °C for 22 cycles. The conditions for semi-quantitative RT-PCR were chosen so that none of the mRNAs analyzed reached a plateau at the end of the amplification cycles, i.e. they were in the exponential phase of amplification, and that the two sets of primers (one set of gene specific primer and the other set for *ACTIN2* gene primers) used in each reaction did not compete with each other. Each set of reactions always included a no-sample negative control. A negative control reaction containing RNA instead of cDNA was performed to rule out genomic DNA contamination in each set of reactions. Appropriate number of cycles was determined by testing the different cycles of 15, 19, 23, 27, and 31 for both core DSB repair gene transcripts and *ACTIN2* (internal control) amplifications. The optimal number of cycles was strictly maintained to be in the same range for the specific mRNA of interest and the internal control, so that both can be measured on the same gel. The same set of samples was amplified at the same time in the presence of the specific primers for DSB repair gene transcripts and the internal control *ACTIN2*. Equal amounts of PCR products were then run on the same 1% agarose gel for comparison. Images of the RT-PCR products in ethidium bromide stained agarose gels were acquired in Bio-Rad Molecular Imager Gel Doc XR system with high resolution CCD camera and quantification of the bands was performed by Bio-Rad Image Densitometer GS-700 using Quantity One software (data not shown). Representative gel images from two independent trials are shown in Fig.S2.

**Supplementary Literature Cited**

1.Roy S, Choudhury SR, Sengupta DN, Das KP (2013) Involvement of AtPolλ in the

Repair of High Salt- and DNA Cross-Linking Agent-Induced Double Strand Breaks in Arabidopsis. Plant Physiol 162: 1195-1210.

**Supplementary Figure legends**

**Supplementary Figure S1.** **Schematic structure of major marker genes involved in the detection, signaling and repair of DNA double strand breaks in *Arabidopsis thaliana*.** The genomic organization of the major DSB related genes including *AtKU80* (AT1G48050), *AtKU70* (AT1G16970), *Atlig4* (AT5G57160), *AtXRCC4* (AT3G23100), *AtPolλ* (At1G10520), *AtMRE11* (AT5G54260), *AtRAD50* (AT2G31970), *AtNbs1* (AT3G02680), *AtRAD51* (AT5G20850), *AtATR* (AT5G40820) have been indicated. The exons are indicated by pink rectangles, while the intron regions are shown with thick blue line. The T-DNA insertions are shown by inverted triangles. Accession numbers of the sequences used in this study have been indicated within parenthesis. The numbers indicate the relative lengths of the genes. For the determination of genomic organization of the genes, an mRNA-to-genomic alignment program was performed using Spidey (<http://www.ncbi.nlm.nih.gov/spidey/>).

**Supplementary Figure S2. Characterization of T-DNA insertion mutant lines for DSB-related genes.** A, Expression of core DSB repair genes **(A)** *AtKU80*, (B) *AtKU70*, (C) *AtLig4*, (D) *AtXRCC4*, (E) *AtPolλ*, (F) *AtATM*, (G) *AtATR*, (H) *AtMRE11*, (I) *AtRAD50*, (J) *AtNbs1*, (K) *AtRAD51* and (L) *AtBRCA1* in 7-days-old wild-type and corresponding mutant *Arabidopsis* seedlings. Lane 1 in each panel indicates mRNA levels of the respective genes under wild-type background while lane 2 indicates expression level in T-DNA knockout mutant background Expression of *ACTIN2* (*ACT2*) has been shown as control (A-L, lower panels). Specificity of amplification of transcripts was confirmed by direct sequencing of the gel purified PCR products.
