# Supplementary material for: Homologous Recombination Defective Arabidopsis Mutants Exhibit Enhanced Sensitivity to Abscisic Acid
Source: PLoS One. 2017 Jan 3;12(1):e0169294. doi: 10.1371/journal.pone.0169294 (PMC5207409; doi:10.1371/journal.pone.0169294)
Supplement: S1 Table — (DOC) [file pone.0169294.s002.doc]

**S1 Table: Primers used for PCR genotyping for the characterization of T-DNA insertion mutant lines in *Arabidopsis thaliana***

| **T-DNA insertion mutant lines from ABRC, Ohio** | **Primer Sequence** |
| --- | --- |
| *Salk_016627C* (*AtKU80* - AT1G48050) | LP 5’ TTGTGACAGCTGTCCAAATTG 3’  RP 5’ ACACGTTTCGTGGAAGTTTTG 3’ |
| *Salk_123114C*  *(AtKU70 - AT1G16970)* | LP 5’ TTACTTTGTTGTTTCGGGTGC 3’  RP 5’ CTCTTGGCAAGTACACGCTTC 3’ |
| *Salk_044027C*  *(AtLig4 - AT5G57160)* | LP 5’ GCTTCAAGTGAGAACAGGTGC 3’  RP 5’ CTGATTCGAACCAAACTCAGC 3’ |
| *Salk_052736C (AtXRCC4–AT3G23100)* | LP 5’ GATCCCTCTAGAGCATCGGAG 3’  RP 5’ TATAAAATCCACTAAGGCGCG 3’ |
| *Salk_075391C, atpolλ-1*  *(AtPolλ - AT1G10520)* | LP 5’ CAATGACCGAACTGGAGCTAG 3’  RP 5’ ATACGTGTCAACGCCTGAATC 3’ |
| *Salk_006953, atm-2*  (AtATM - AT3G48190) | LP 5’ CTTCACACAGAGGATCTTCGC 3’  RP 5’ GCAATGCTTAGTCGCTTTCAG 3’ |
| *Salk_032841*  *(AtATR -* *AT5G40820)* | LP 5’ GCAGCAAAAATTTCTTGGTTG 3’  RP 5’ ACTTCAAGGGTTCCGATGTTC 3’ |
| *Salk_054418, atmre11-3*  *(AtMRE11 - AT5G54260)* | LP 5’ TTCTGGTGTTGGCCAGATTAC 3’  RP 5’ CCAATGGGAGTTTGATCTCTG 3’ |
| *Salk_084967*  *(AtRad50 - AT2G31970)* | LP 5’ CCGAGAAGGATATGAAGAGGG 3’  RP 5’ TCAAAAACTGCCCGTAGTTTG 3’ |
| *SALK_057446C*  *(AtNBS1 - AT3G02680)* | LP 5’ GAATGCAGCGAAGAAGATGAG 3’  RP 5’AAACCCAAGAAGAAATGTGGG 3’ |
| *Sail_873_C08*  (*AtRad51 – AT5G20850)* | LP 5’ TTCAGGATGGTGTCTCAGAGC 3’  RP 5’ ATGCCAAGGTTGACAAGATTG 3’ |
| *Salk_014731*  *(AtBRCA1 - At4G21070)* | LP 5’ CAAAGAGTCGCTTTGTTCCTG 3’  RP 5’ TATCACTTGCCTTTTCAACGG 3’ |
| *Salk_089362* (*AtRAD52 - AT1G71310*) | LP 5’ GAAAGACCTCAGCATCAGTGC 3’  RP 5’ CAAGCACTTTCGTTTTCAGATG 3’ |
| *Salk_038057* (*AtRAD54 - AT3G19210*) | LP 5’ TTACTCAAGTTTCCTTGGGGG 3’  RP 5’ CTCGTTGAGAGATAACGGCTG 3’ |
