# Supplementary material for: Homologous Recombination Defective Arabidopsis Mutants Exhibit Enhanced Sensitivity to Abscisic Acid
Source: PLoS One. 2017 Jan 3;12(1):e0169294. doi: 10.1371/journal.pone.0169294 (PMC5207409; doi:10.1371/journal.pone.0169294)
Supplement: S2 Table — (DOC) [file pone.0169294.s003.doc]

**S2 Table: Primer sequences used for transcript profile analysis by quantitative real-time PCR**

| **Gene name** | **Primer Sequence** | **Annealing temperature (°C)** |
| --- | --- | --- |
| ***AtKU80* (AT1G48050)** | |  | Fw 5’[TCCTGCGATGCGTAGTGTTCTC](about:blank) 3’ | |  | | --- | --- | --- | --- | | Rev 5’[AGCAAGCTCGTTTCCAGTTTCTTC](about:blank) 3' | |  | | | 63 |
| *AtKU70* (AT1G16970) | Fw 5’[AGCAGTCGATTTATGGCGATGACC](about:blank) 3’  Rev 5’[CCACAGTCAAGTCCTTCAGCTTTC](about:blank) 3’ | 62 |
| *AtLig4* (AT5G57160) | Fw 5’TTGGCTTCAAGTGAGAACAGAGC 3’  Rev 5’TGACCCACTTCATCTCCTGAGC 3’ | 63 |
| *AtXRCC4*  (AT3G23100) | Fw 5’CGAAACACACTTGTCTCCGTCTCG 3’  Rev 5’CGTCGCATTGCAAATCCAAGAGG 3’ | 65 |
| *(AtPolλ* (AT1G10520) | Fw 5’[ACGACGTGTTGAACAGAAGGC](about:blank) 3’  Rev 5’[AGCTGGGAATAGTCCAGTGTCG](about:blank) 3’ | 60 |
| *AtATM-* (AT3G48190) | Fw 5’TTGGTCTTGGTGACCGACATGC 3’  Rev 5’TCTTGTCAGTCTGAACGGAACCC 3’ | 63 |
| *AtATR* (AT5G40820) | Fw 5’GTGCCATTCAGATTGACCCAGAAC 3’  Rev 5’TGCCCTCATATCCAGTGATGCC 3’ | 65 |
| *AtMRE11* (AT5G54260) | Fw 5’AACAAATCTCAGCCTCGGGTTAC 3’  Rev 5’AGAAGTTGTTCCGCTTGAGAGGTC 3’ | 60 |
| *AtRad50* (AT2G31970) | Fw 5’CCCGCTCTTACAGCTACAAGGTTC 3’  Rev 5’TTGACCTGCACTGCATCTTCCTC 3’ | 65 |
| *AtNBS1* (AT3G02680) | Fw 5’CTTCACTGATACCACCATCCGTTG 3’  Rev 5’GCTTCAGAATCCGCTACCACTG 3’ | 65 |
| *AtRad51* (AT5G20850) | Fw 5’TTCCGCTCTGGAAAGACTCAGC 3’  Rev 5’ACCTCCTTGATCCATGGGAAGTTG 3’ | 63 |
| *AtBRCA1* (At4g21070) | Fw 5’TGCATCCATTAAGTTGCCCTGTG 3’  Rev 5’TAGGCTGAGAGTGCAGTGGTTC 3’ | 62 |
| *AtRAD52* (*AT1G71310*) | Fw 5’ GCTCCATCAAATACATCCCTTGGC 3’  Rev 5’TCAGTGCCGTAGAGAGTCACAC 3’ | 63 |
| *AtRAD54* (*AT3G19210*) | Fw 5’ TCATGGTGCATTCAGTGCTTGTC 3’  Rev 5’ CATGAACTGGACACCCTCTCTTTG 3’ | 63 |
| *ACTIN2* | Fw 5’GTTGAACGGAAGGGATTGAGAGT 3’  Rev 5’AAAACCACTTACAGAGTTCGTTCG 3’ | 63 |
