# Supplementary material for: Homologous Recombination Defective Arabidopsis Mutants Exhibit Enhanced Sensitivity to Abscisic Acid
Source: PLoS One. 2017 Jan 3;12(1):e0169294. doi: 10.1371/journal.pone.0169294 (PMC5207409; doi:10.1371/journal.pone.0169294)
Supplement: S1 Fig — (PPT) [file pone.0169294.s004.ppt]

## Slide 1
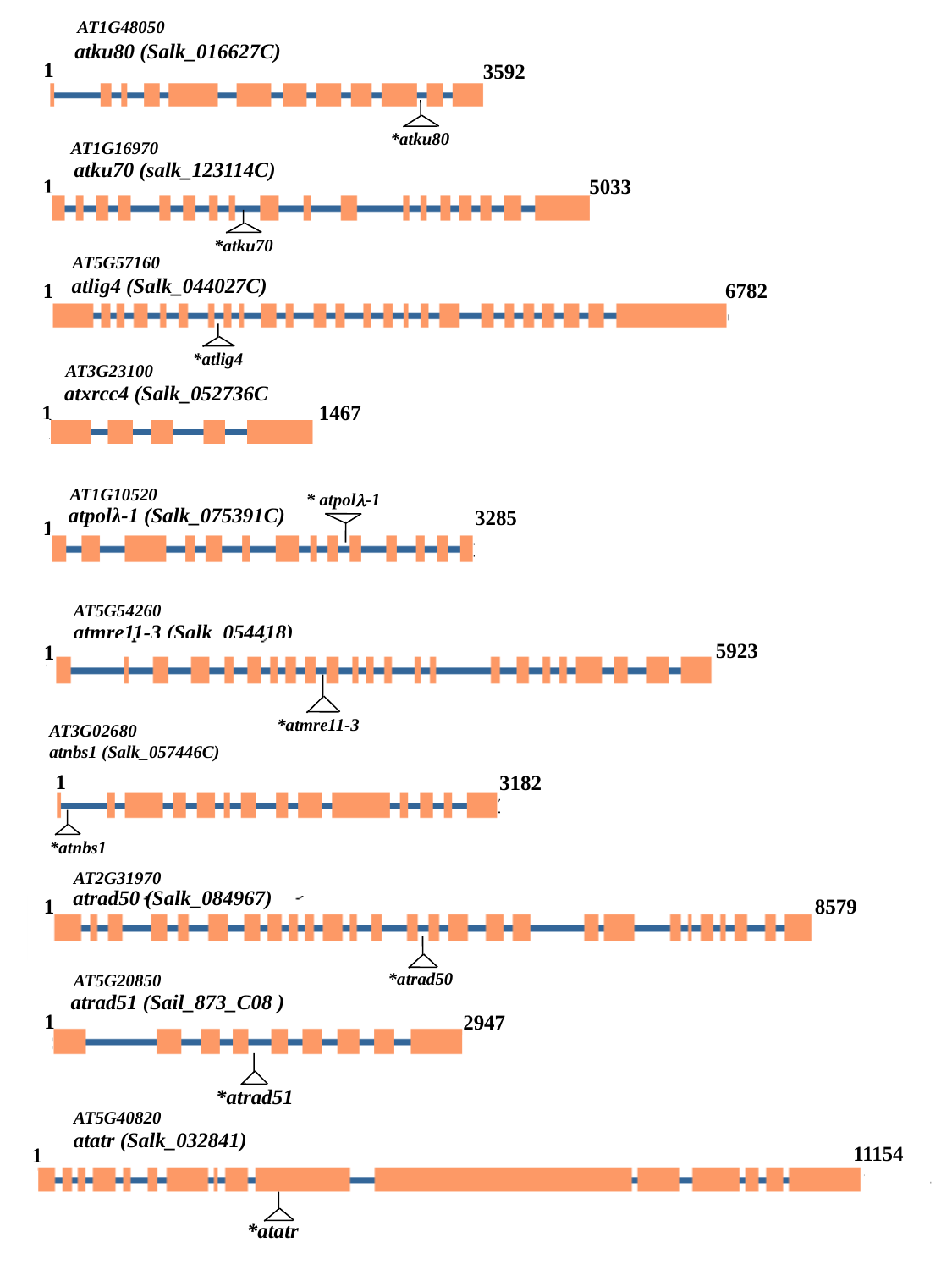

AT1G48050
atku80 (Salk_016627C)
1
3592
*atku80
AT1G16970
atku70 (salk_123114C)
1
5033
*atku70
AT5G57160
atlig4 (Salk_044027C)
6782
1
*atlig4
AT3G23100
atxrcc4 (Salk_052736C
1
1467
AT1G10520
* atpolλ-1
atpolλ-1 (Salk_075391C)
3285
1
AT5G54260
atmre11-3 (Salk_054418)
5923
1
*atmre11-3
AT3G02680
atnbs1 (Salk_057446C)
1
3182
*atnbs1
AT2G31970
atrad50 (Salk_084967)
1
8579
*atrad50
AT5G20850
atrad51 (Sail_873_C08 )
1
2947
*atrad51
AT5G40820
atatr (Salk_032841)
11154
1
*atatr

## Slide 2
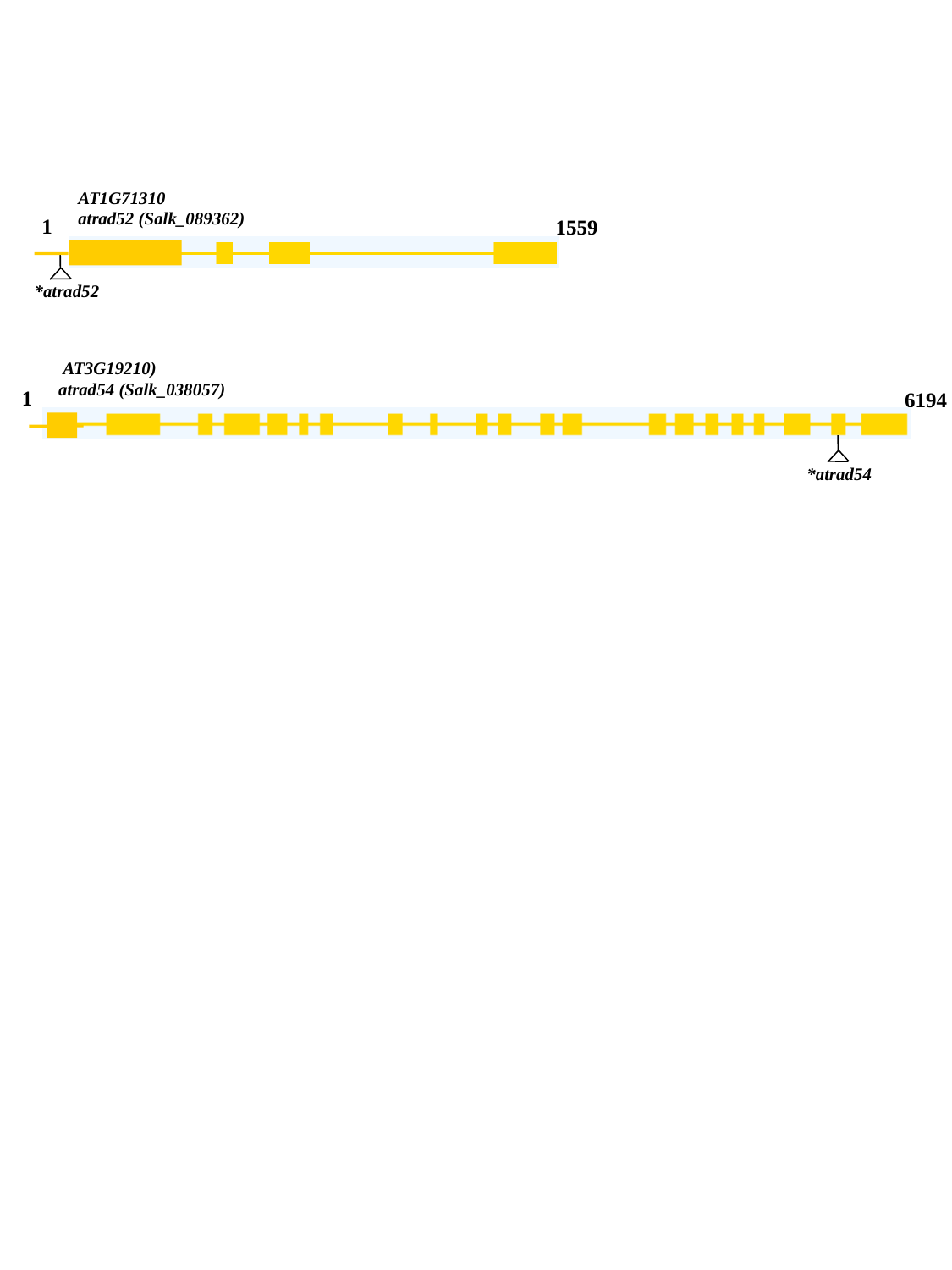

AT1G71310
atrad52 (Salk_089362)
1
1559
*atrad52
 AT3G19210)
atrad54 (Salk_038057)
1
6194
*atrad54
