# Supplementary material for: Homologous Recombination Defective Arabidopsis Mutants Exhibit Enhanced Sensitivity to Abscisic Acid
Source: PLoS One. 2017 Jan 3;12(1):e0169294. doi: 10.1371/journal.pone.0169294 (PMC5207409; doi:10.1371/journal.pone.0169294)
Supplement: S2 Fig — (PPT) [file pone.0169294.s005.ppt]

## Slide 1
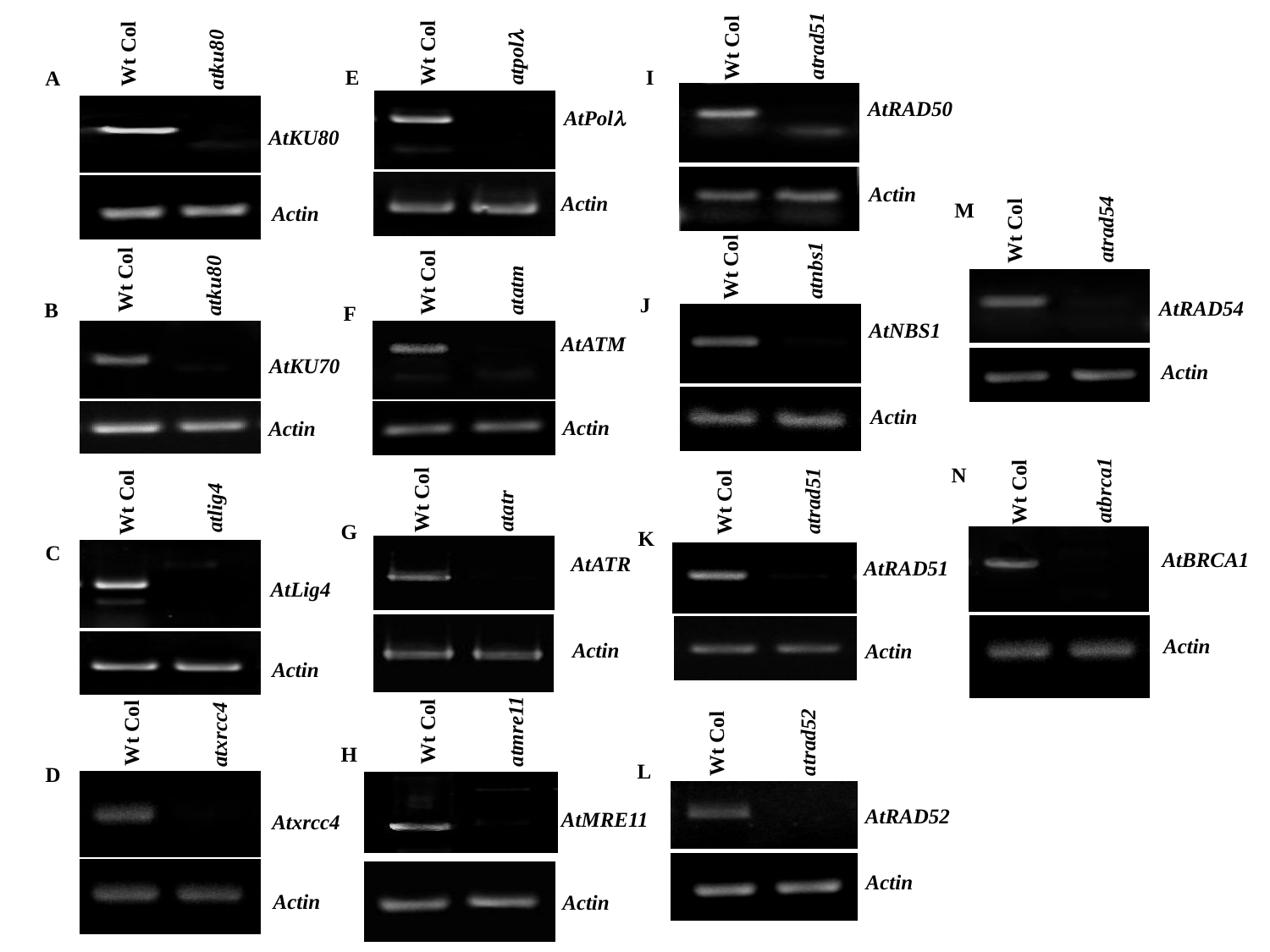

atrad51
Wt Col
AtRAD50
Actin
Wt Col
Wt Col
atpol
atku80
E
I
A
AtPol
AtKU80
atrad54
Wt Col
AtRAD54
Actin
Actin
M
Actin
Wt Col
atnbs1
AtNBS1
Actin
Wt Col
Wt Col
atku80
atatm
J
B
F
AtATM
AtKU70
Actin
Actin
atbrca1
Wt Col
AtBRCA1
Actin
N
atrad51
Wt Col
AtRAD51
Actin
Wt Col
atatr
AtATR
Actin
Wt Col
atlig4
G
K
C
AtLig4
Actin
atmre11
Wt Col
AtMRE11
Actin
Wt Col
atxrcc4
atrad52
Wt Col
H
L
D
AtRAD52
Atxrcc4
Actin
Actin
